# Interlaboratory study for the evaluation of three microtiter plate-based biofilm quantification methods

**DOI:** 10.1038/s41598-021-93115-w

**Published:** 2021-07-02

**Authors:** Jontana Allkja, Frits van Charante, Juliana Aizawa, Inés Reigada, Clara Guarch-Pérez, Jesus Augusto Vazquez-Rodriguez, Paul Cos, Tom Coenye, Adyary Fallarero, Sebastian A. J. Zaat, Antonio Felici, Livia Ferrari, Nuno F. Azevedo, Albert E. Parker, Darla M. Goeres

**Affiliations:** 1 LEPABE - Laboratory for Process Engineering, Environment, Biotechnology and Energy, Faculty of Engineering, University of Porto, Rua Dr. Roberto Frias, 4200-465 Porto, Portugal; 2 Center for Biofilm Engineering, Montana State University, 366 Barnard Hall, Bozeman, MT 59717 USA; 3 Laboratory of Pharmaceutical Microbiology, Ghent University, Ghent, Belgium; 4 Laboratory for Microbiology, Parasitology and Hygiene (LMPH), Faculty of Pharmaceutical, Biomedical and Veterinary Sciences, University of Antwerp, Antwerp, Belgium; 5 Drug Research Program, Division of Pharmaceutical Biosciences, Faculty of Pharmacy, University of Helsinki, 00790 Helsinki, Finland; 6 Department of Medical Microbiology and Infection Prevention, Amsterdam Institute for Infection and Immunity, Amsterdam UMC, University of Amsterdam, 1105 AZ Amsterdam, The Netherlands; 7 Discovery Microbiology, Aptuit (Verona) S.R.L., An Evotec Company, Verona, Italy; 8 ESCMID Study Group for Biofilms, Basel, Switzerland; 9 Department of Mathematical Sciences, Montana State University, Bozeman, MT USA

**Keywords:** Biofilms, Microbiology techniques, Applied microbiology

## Abstract

Microtiter plate methods are commonly used for biofilm assessment. However, results obtained with these methods have often been difficult to reproduce. Hence, it is important to obtain a better understanding of the repeatability and reproducibility of these methods. An interlaboratory study was performed in five different laboratories to evaluate the reproducibility and responsiveness of three methods to quantify *Staphylococcus aureus* biofilm formation in 96-well microtiter plates: crystal violet, resazurin, and plate counts. An inter-lab protocol was developed for the study. The protocol was separated into three steps: biofilm growth, biofilm challenge, biofilm assessment. For control experiments participants performed the growth and assessment steps only. For treatment experiments, all three steps were performed and the efficacy of sodium hypochlorite (NaOCl) in killing *S. aureus* biofilms was evaluated. In control experiments, on the log_10_-scale, the reproducibility SD (S_R_) was 0.44 for crystal violet, 0.53 for resazurin, and 0.92 for the plate counts. In the treatment experiments, plate counts had the best responsiveness to different levels of efficacy and also the best reproducibility with respect to responsiveness (Slope/S_R_ = 1.02), making it the more reliable method to use in an antimicrobial efficacy test. This study showed that the microtiter plate is a versatile and easy-to-use biofilm reactor, which exhibits good repeatability and reproducibility for different types of assessment methods, as long as a suitable experimental design and statistical analysis is applied.

## Introduction

The lack of reproducibility among published studies has become one of the bigger concerns across different scientific research areas and biofilm research is certainly no exception^1,2^. This is due to many different factors, including selective or incomplete reporting of the experiments performed and lack of robust methods^3,4^.

Microtiter plate-based methods are some of the more widely used methods in biofilm research. They are inexpensive, easy to use, versatile and adaptable for high-throughput screening^5,6^. However, little is known about their repeatability (within lab variation) and there are currently no reports on their reproducibility (among lab variation)^7,8^. The microtiter plate is a batch system in which the biofilm is grown in a small volume and then assessed using a variety of methods. Most assessment methods rely on the use of dyes (e.g. crystal violet and resazurin) to stain the biofilm or some of its components; colony forming unit (CFU) counts are also used^9^. The variety of assessment methods available has led to many possible applications for microtiter plate-based methods such as antimicrobial compound screening, assessing the biofilm forming ability of different microorganisms, and evaluating the effect of various factors on biofilm formation^10–13^.

Considering the widespread use of microtiter plate methods in the field, the evaluation of their reproducibility is of a clear interest to the biofilm community as it would provide an insight into the potential of microtiter plates for standardisation, which could be useful for antimicrobial screening tests^14^. Additionally, it could serve as a starting point for making microtiter plate data comparable across research groups and also help us better understand what factors might affect the performance of microtiter plate experiments.

Efforts have started to improve the reporting of microtiter plate experiments, through the implementation of minimum information guidelines on reporting biofilm experiments (www.miabie.org)^15,16^. Moreover, the repeatability and reproducibility of methods using other biofilm reactors have been evaluated, which can serve as a reference for evaluating microtiter plate methods. Devices such as the MBEC, the CDC Biofilm Reactor and Drip Flow Biofilm Reactor were assessed through interlaboratory studies, where 6–8 different laboratories perform the same protocol^17–19^. As a result of these tests, several standard methods for the use of these devices in antimicrobial testing have been approved^20–22^. In order for a protocol to be accepted as a standard method, the standard deviation (SD) of the reproducibility needs to be “small”, often determined by standard methods societies, e.g., American Society for Testing and Materials (ASTM).

In the present study, we aim to evaluate the reproducibility and responsiveness of three different biofilm assessment methods (crystal violet, resazurin and plate counts) applied to microtiter plates through a ring trial, performed across five different laboratories located in the US and Europe. This paper reports on the repeatability in each lab and the reproducibility across different labs of each of these three assessment methods. Additionally, it evaluates the responsiveness of these methods in assessing an antimicrobial efficacy test.

## Materials and methods

The description provided in this “Materials and methods” section complies with the recently-published guideline on reporting spectrophotometric and fluorometric methods to assess biofilms in microplates^16^.

### Study design

The ring trial investigated three different biofilm assessment methods in flat bottom, untreated, polystyrene, 96-well microtiter plates: plate counts, resazurin and crystal violet. Six laboratories initially took part and are identified by numerical Lab IDs between 1 and 6. One participant from each laboratory performed the experiments. The participants were provided with a detailed inter-lab protocol (ILP) and a list of supplies (S1 supplementary information file). The protocol was tested and optimized prior to the start of the interlaboratory study. ILP was divided into three distinct steps: biofilm growth, biofilm challenge and biofilm assessment. For the latter, separate protocols for each assessment method were provided. Participants were instructed to find closest substitute/match for materials in the supply list (S1 Supplementary Information File) when possible, hence there is a difference in supplier between labs.

The data collected from Lab 5 have been removed from all data analysis. An initial analysis identified their control data as severe outliers. Subsequent inquiries revealed that their treatment experiments data were also unusable due to deviations from performing the protocols and a lack of documentation. Results of control data analysis with Lab 5 data included can be found in S2 supplementary information file.

#### Biofilm growth

*Staphylococcus aureus subsp. aureus* (ATCC 25923) was used for the ring trial. Briefly, stocks were streak plated on Tryptic Soy Agar (TSA) at 37 ± 2 °C for 24 h. One or two colonies were transferred into 15 mL Tryptic Soy Broth (TSB) and incubated at 37 ± 2 °C, 125 rpm, overnight. An aliquot was sub-inoculated in fresh TSB at 37 ± 2 °C, 125 rpm until exponential growth phase [OD = 0.300 (595 nm) or 7.5 ± 0.5 Log_10_ CFU/mL] was achieved. This inoculum was diluted to a concentration of 5.5 ± 0.5 Log_10_ CFU/mL. 200 µL/well was transferred to a 96-well plate for biofilm formation at 37 ± 2 °C, no shaking for 24 h in a humidified incubator. Wells containing only TSB were used as negative controls to check for contamination. The full biofilm growth protocol can be found in S1 supplementary information file.

#### Plate reader tests

Crystal violet and resazurin require the use of plate readers and output values can vary greatly across different models and manufacturers. Hence, calibration curves were necessary to compare the outputs from different labs. For crystal violet different concentrations of the dye were added to the wells of a microtiter plate (starting from 0.01 g/L) and their absorbance at λ = 595 nm was measured. For resazurin, the participants chemically reduced resazurin into resorufin and added different concentrations to a microtiter plate (starting from 5 µg/mL) and measured fluorescence at λ_excitation_ = 560 nm; λ_emission_ = 590 nm. The detailed protocol can be found in S1 supplementary information file.

#### Control experiments

For the control experiments, only the biofilm growth and biofilm assessment parts of the protocol were performed. One 96-well plate was used per assessment method, per experimental day, two experimental days in total. Due to the nature of the methods for plate counts only 15 wells/plate were quantified, whereas for crystal violet and resazurin the entire plate (minus control wells) was assessed. Laboratories with their own in-house protocols (IHP) for the assessment methods prepared an extra plate and performed their own assessment protocol in parallel with the ILP used for the ring trial. For both plates the biofilm was prepared using the ILP biofilm growth protocol. The detailed protocols are found in the S1 Supplementary Information File.

#### Treatment experiments

For the treatment experiment, all three steps of the ILP were performed. For the biofilm challenge step, sodium hypochlorite (NaOCl), i.e., bleach, was used to challenge the biofilm. Half of one 96-well plate was used per method, per experimental day, with three experimental days in total. Titration tests were used to measure the total concentration of chlorine (Cl). The antimicrobial was then diluted to four different concentrations of Cl in NaOCl reported as NaOCl concentrations (1000 mg/L, 500 mg/L, 100 mg/L and 10 mg/L) which, were added to the biofilm (200 μl/well) for 10 min and neutralised by washing twice with PBS. Ultra-pure water was added to the controls to account for the effect of the fluid exchange on biofilm removal. All concentrations were tested on the same day, 8 wells per treatment. For plate counts only 3 wells per concentration per plate were quantified, for crystal violet and resazurin 8 wells per concentration per plate were assessed. No IHP treatment data were generated; IHP data were generated only for control experiments. Detailed protocols are found in the S1 supplementary information file.

### Statistical analysis

#### Plate count data treatment

The colonies formed were counted on each plate, averaged across plates, multiplied by the dilution factor 10^d^, divided by the drop volume (10 µL), multiplied by the total well volume (200 µL) and then converted to a log density (LD) per well using the following equation:

$${\text{LD }} = {\text{ log}}_{{{\text{1}}0}} \left( {{\text{CFU }}/{\text{ well}}} \right){\text{ }} = {\text{ log}}_{{{\text{1}}0}} [({\text{1}}0^{{\text{d}}} \times {\text{ }}\left( {{\text{average CFU}}} \right){\text{ }}/{\text{ 1}}0\mu {\text{L}}){\text{ }} \times {\text{ 2}}00\mu {\text{L}}]$$

For the treatment experiments when no colonies were present on agar plates, a value of 0.5 CFU/well was assigned to a single plate at the lowest dilution counted. The antimicrobial’s effect was quantified as a log reduction (LR). A single LR value was calculated for each chlorine concentration used, for each experimental day. The LR is equal to the mean of the treated log density (LD) for each treatment concentration subtracted from the mean of the control LD.

#### Crystal violet and resazurin data transformation

“Raw” data collected from the plate reader tests were used to generate calibration curves for each method and each lab. Equations were generated for these curves by linear regression analysis and they were used to transform the data. Based on the regression analysis, log_10_ fluorescence vs log_10_ resorufin was chosen as the calibration curve for the resazurin data as it better fit the initial calibration data and allowed for better prediction for resazurin/resorufin concentrations from the later part of the study that fell outside the range of the calibration curve. This extrapolation occurred because the resazurin was chemically reduced for the calibration phase of the study making it difficult to ensure that the fluorescence values used to generate the calibration curve contained the values measured during the later biofilm experimentation phase. This extrapolation is a potential weakness of our results. In summary, “raw” fluorescence data were transformed to log_10_ fluorescence and further transformed to log_10_ resorufin concentration (µg/mL) based on the calibration curve equation for each lab.

For crystal violet, absorbance vs crystal violet concentration was chosen as the calibration curve. Hence, “raw” optical density data were transformed to crystal violet concentrations (µg/mL) based on the calibration curve equation for each lab. Although initial experiments were performed at a single lab to estimate the CFUs corresponding to crystal violet concentrations at that lab, each lab did not generate such a calibration curve, so it is not possible to transform the ODs or crystal violet concentrations to CFU densities.

For both methods, negative control values (wells containing TSB only, no bacteria) were not taken into consideration as there were no negative controls in the plate reader test, so the calibration curves do not account for negative controls. Calibration curve plots and equations can be found in supplementary information file S3.

#### Repeatability and reproducibility assessment

To investigate the repeatability (day-to-day variability) and reproducibility (lab-to-lab variability) for the control data generated by the methods, the data were first log_10_-transformed: LD for plate counts, log_10_ resorufin or log_10_ crystal violet concentration. A mixed effects ANOVA model was then fit to the log-transformed responses for each of the 3 methods separately. ‘Lab ID’ and ‘day’ were assigned as random factors, with ‘day’ nested within ‘Lab ID’, meaning the levels of the factor ‘day’ are different for each lab. The model estimates the variability in the response for three different sources: error (well-to-well), day (day-to-day) and lab (lab-to-lab). The repeatability variance of the method is represented by the error + day variance. The repeatability standard deviation (S_r_) is the square root of this variance. The reproducibility variance of the method is represented by the sum of all variance components and the reproducibility SD (S_R_) is the square root of this variance^23^. It is common to report the variance components as percentages of the “total” reproducibility variance (see e.g. ASTM E691) calculated by dividing each variance component by the reproducibility variance.

For the treatment experiments, the reproducibility SD of LRs was estimated for each NaOCl concentration separately by fitting a mixed effects ANOVA to the LRs for each method separately with a random factor for Lab ID.

#### ILP versus IHP comparison

To evaluate the effect that the change of protocol (ILP vs IHP) had on the control data we fit a mixed effects ANOVA to the log responses for all 3 methods where Day and Lab ID were assigned as random factors and protocol was assigned as a fixed factor. The ANOVA was followed by Tukeys 95% CIs to determine statistical equivalence at 97.5% confidence for the median responses for ILP and IHP (CFU density for plate counts, resoforin concentration, and crystal violet). For the plate count method the equivalency margin of 0.5 logs was chosen based on previous examples in the literature^24,25^. On the other hand, no previous reference was found for establishing an equivalency margin for resazurin and crystal violet method, so the equivalency margin of 0.5 log was used for these methods as well. When comparing IHP vs ILP for each of the 3 methods, the equivalency margin of 0.5 applied on the log scale means that differences in means of the log-transformed responses up to 0.5 log are negligible and not of practical importance. When antilogging, the conclusion is that differences up to a 70% decrease (1 − 10^–0.5^) or a threefold increase (10^0.5^) in the median response for the IHP compared to median response for the ILP are negligible and not of practical importance.

#### Responsiveness assessment

To assess the responsiveness of the methods to the NaOCl treatment we constructed dose–response curves using the mean LR concentrations per NaOCl concentration, per day, per lab. A mixed effects ANOVA with a random factor for Lab ID and Log_10_ NaOCl as a covariate was fit to the LRs for all concentrations to assess the responsiveness of the method and also to assess the reproducibility pooled across all concentrations.

Furthermore, a mixed effects ANOVA was performed for each NaOCl concentration separately and the resulting S_R_ values were fit by a parabola as a function of LR. To enforce our prior belief that the parabola should be concave, the leading term was constrained to be non-positive^26^. This constraint only had to be enforced for crystal violet data, where the leading term was set to zero.

#### Model assessment and software

The choice to log-transform the responses (CFU density for the plate count data, resorufin concentration for the resazurin data, crystal violet concentration) is crucial. Initially, for control and efficacy responses each of the 3 methods, the log-transform was required to satisfy the ANOVA assumptions of normality and constant variance. These assumptions were checked using “normal probability” and “residual vs fits” plots of the residuals. Just as importantly, having all responses on the log-scale made method comparisons straightforward. Because SDs of log responses are invariant to a change in units, here we directly compare the SDs from the different methods. Furthermore, because a difference in log responses such as a LR is unitless, we directly compare slopes and the mean LR values when assessing the responsiveness of the methods.

All statistical assessments were performed using Minitab v.18® [Minitab 18 Statistical Software (2019). State College, PA: Minitab, Inc. (www.minitab.com)] Mixed Effects Models Functionality that allows the user to specify (as described above): the response (control or LR), fixed effects (e.g., ILP vs IHP), random effects (lab and day nested in lab) and covariate (e.g., time). From the full data set supplied via the link in the Supplementary Materials, Minitab’s Mixed Effects Model function, with the inputs just described, could be used to reproduce the results that we present here. Minitab was also used to generate all the figures. Horizontal jitter was applied to the points in the graphs to better visualise the data.

## Results

### Control

#### Interlaboratory protocol analysis

**Figure 1:**
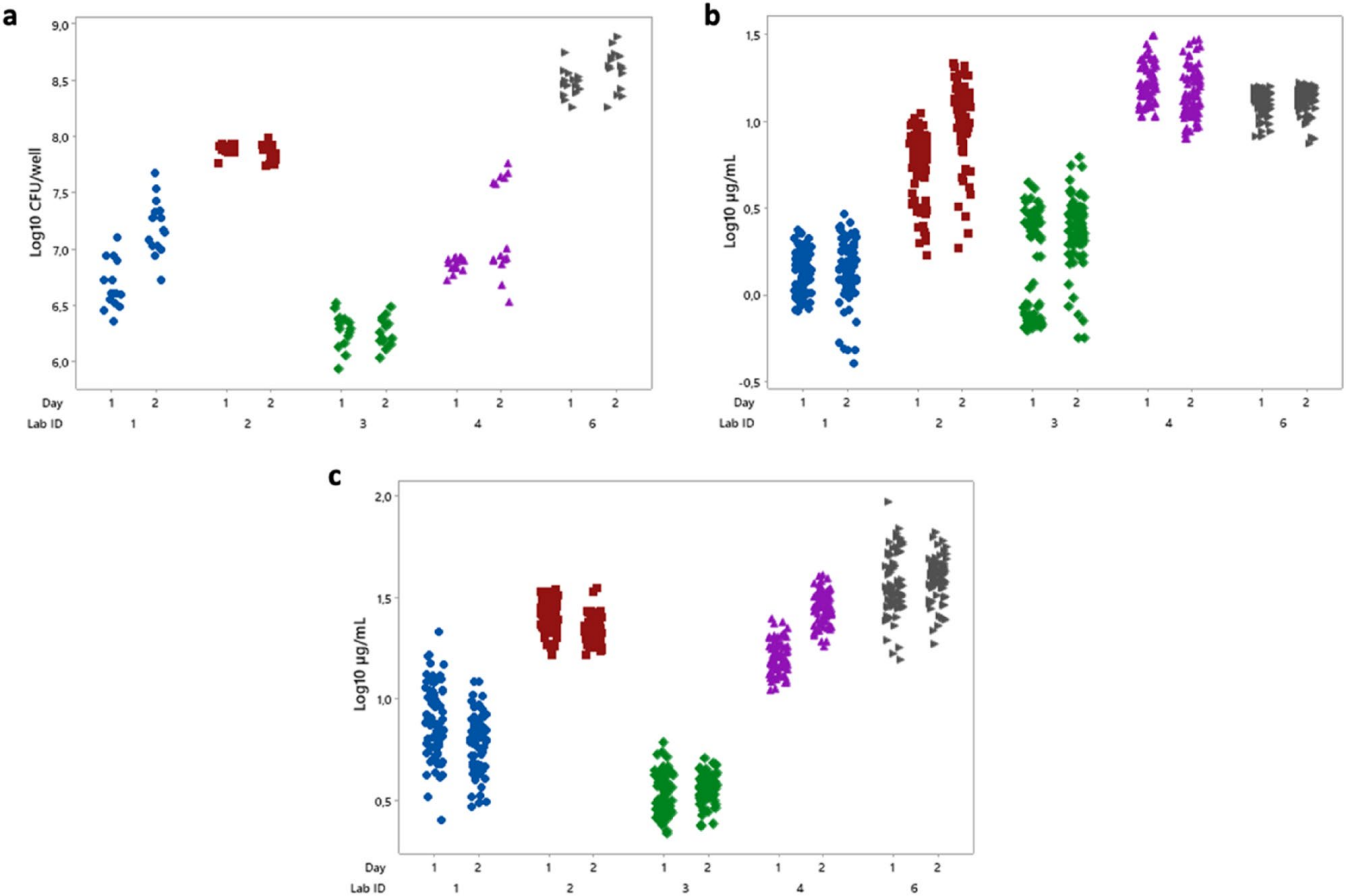
Control experiment data for the ILP protocol. Along the horizontal axis are listed the lab IDs and the two experimental days within each lab. Horizontal jitter has been applied to better visualize data points. (**A**) Plate count—Each point in the graph is the log density (LD = log10(CFU/well)) of biofilm bacteria grown on a single well. (**B**) Resazurin—Each point in the graph is the log_10_ resorufin concentration (µg/mL) of biofilm bacteria grown on a single well. (**C**) Crystal violet—Each point in the graph is the log_10_ crystal violet concentration (µg/mL) of biofilm bacteria grown on a single well.

To investigate the repeatability and reproducibility of the control experiments for the ILP protocols, the data collected for each lab, across two experimental days, have been included in Fig. 1. A summary of the results can be found in Table 1. From Fig. 1A and Table 1 we can see that most of the variability is due to lab-to-lab differences. Similar to the plate count method, the variability of resazurin method (Fig. 1B) and crystal violet method (Fig. 1C) is mostly due to lab-to-lab differences. The trend of the data is similar across all three methods (Fig. 1), meaning labs that respond lower for one method do so for the other two as well e.g., lab 3 has the lower values on average for all 3 methods compared to the rest of the labs. Likewise, the labs with higher responses for one method, have higher responses for the other methods as well e.g., lab 6.

**Table 1:** Summary of statistical analysis for the ILP control data for each method.

Method	Mean log_10_ response ± SE	Units	Variance components		Standard deviation	
			day + error	Lab	Repeatability	Reproducibility
Plate count	7.32 ± 0.40	CFU/well	8.82%	91.18%	0.27	0.92
Resazurin	0.71 ± 0.22	µg/mL	15.89%	84.11%	0.21	0.53
Crystal Violet	1.13 ± 0.19	µg/mL	10.18%	89.82%	0.14	0.44

The table shows the main conclusions (mean Log values ± Standard Error (SE), variance and standard deviations for repeatability and reproducibility) for the control data using the ILP protocols only for all participating labs, excluding lab 5. Recall from the glossary and methods section that the Repeatability SD is the square root of the “Day + Error” variance, and the Reproducibility SD is the square root of the “total variance”.

When comparing the repeatability and reproducibility across all three methods based on the SD values in Table 1, the crystal violet method is most repeatable and the most reproducible of the three, followed by resazurin and finally the plate count method. As discussed in the Methods, it is possible to directly compare the SDs for the different methods in Table 1 because SDs of log responses are invariant to a change in units.

#### Interlaboratory protocol vs in-house protocol

One of the questions we wanted to answer in the present study was whether the protocol used for the assessment method made a difference in method outcomes. For this purpose, some of the labs, with established in-house protocols (IHP), performed them in parallel to the ILP protocol. For the plate count method only labs 2 and 6 performed IHPs (Fig. S4.1). For the resazurin method, labs 4 and 6 performed IHPs (Fig. S4.2) and for the crystal violet method labs 2, 4, and 6 performed IHPs (Fig. S4.3).

A summary of the statistical comparison of ILP vs IHP for each three methods is given in Table 2. For each method, the ILP protocol has a better (smaller) reproducibility S_R_. However, the mean LDs, log_10_ res and log_10_ CV between the ILP and IHP for each of the three methods were statistically significantly equivalent^27^.

**Table 2:** Comparison of the ILP and IHP control data for each method.

Method	Protocol	Mean log_10_ response ± SE	Units	Standard deviation		ILP IHP	Result
				Repeatability	Reproducibility		
Plate count	ILP	8.20 ± 0.33	CFU/well	0.13	0.49	0.035	Equivalent
	IHP	8.17 ± 0.42	CFU/well	0.17	0.60		
Resazurin	ILP	1.15 ± 0.04	µg/mL	0.11	0.11	0.105	Equivalent
	IHP	1.05 ± 0.04	µg/mL	0.16	0.16		
Crystal Violet	ILP	1.42 ± 0.08	µg/mL	0.14	0.18	− 0.095	Equivalent
	IHP	1.52 ± 0.23	µg/mL	0.19	0.42		

The table shows the main conclusions (mean Log_10_ values ± Standard Error (SE), and standard deviations for repeatability and reproducibility, difference between means, and equivalency testing at 97.5% confidence with an equivalency margin of 0.5 logs) for the control experiments data using the ILP and IHP protocols.

### Treatment

**Figure 2:**
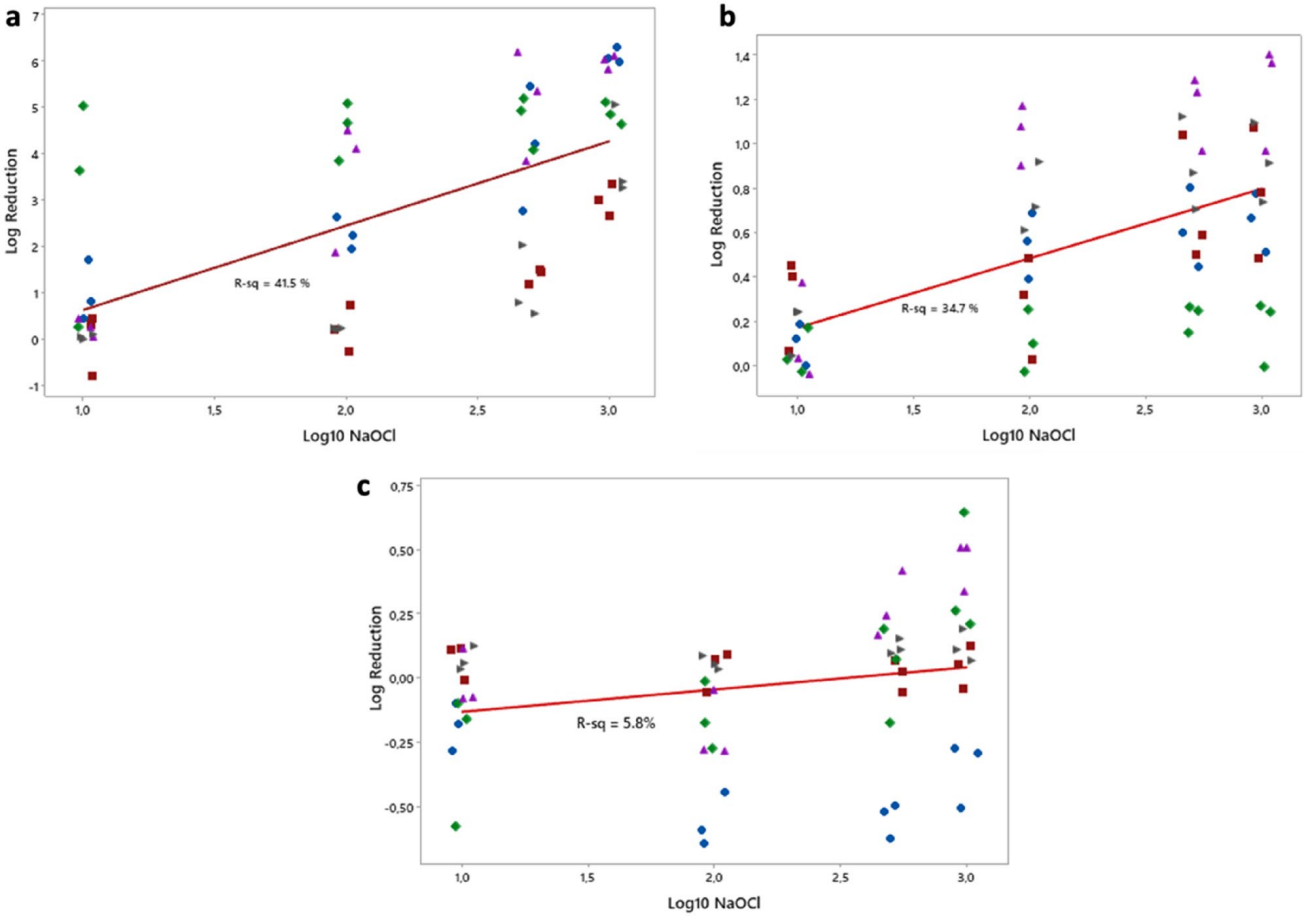
Dose response curves for treatment data. Each data point represents the mean LR per experimental day, per lab. The red curve indicates the regression line. (**A**) Plate count, (**B**) Resazurin, (**C**) Crystal violet.

The dose response curves with respect to the LRs are shown per lab in Figs. S4.4, S4.5 and S4.6. The dose–response curves pooled over all labs are shown in Fig. 2.

A summary of the analysis is shown in Table 3. Based solely on the reproducibility S_R_ values from the table, crystal violet is the most reproducible method with an S_R_ = 0.28, followed by resazurin (S_R_ = 0.36) and plate counts (S_R_ = 1.79).

**Table 3:** Summary of the treatment data for each method.

Method	S_R_	Equation	Slope/S_R_
Plate count	1.79	LR = − 1.196 + 1.823Log_10_NaOCl	1.02
Resazurin	0.36	LR = − 1.406 + 0.3132Log_10_NaOCl	0.87
Crystal Violet	0.28	LR = − 0.2186 + 0.08669Log_10_NaOCl	0.31

The table shows the S_R_ values for the LRs pooled over all concentrations of the NaOCl treatment when applying the ILP protocols only. In addition, the equation of the regression line (shown in Fig. 2) that quantifies the dose response curve for each method. The slope in each equation quantifies responsiveness of each method, hence the column Slope/S_R_ is a measure of the reproducibility of each method relative to the responsiveness.

However, the dose–response for the crystal violet method (Fig. 2C) is quite different compared to the other two. Furthermore, in some instances crystal violet showed negative LR values for the treatment experiments (Fig. 2C and Fig. S4.6), which means that the treated biofilms had higher crystal violet concentrations than the side-by-side controls suggesting that there was an issue with the method despite the low S_R_ value. From the dose response relationship for each method in Table 3, the responsiveness of the method was evaluated based on the slope of the regression line. The larger the slope, the more responsive the method is. Hence, the plate count method is the most responsive of the three, and crystal violet was the least responsive. As discussed in the “Methods”, it is possible to directly compare the mean LRs, SDs and slopes for the different methods because LRs, SDs and slopes of log responses are invariant to a change in units.

**Figure 3:**
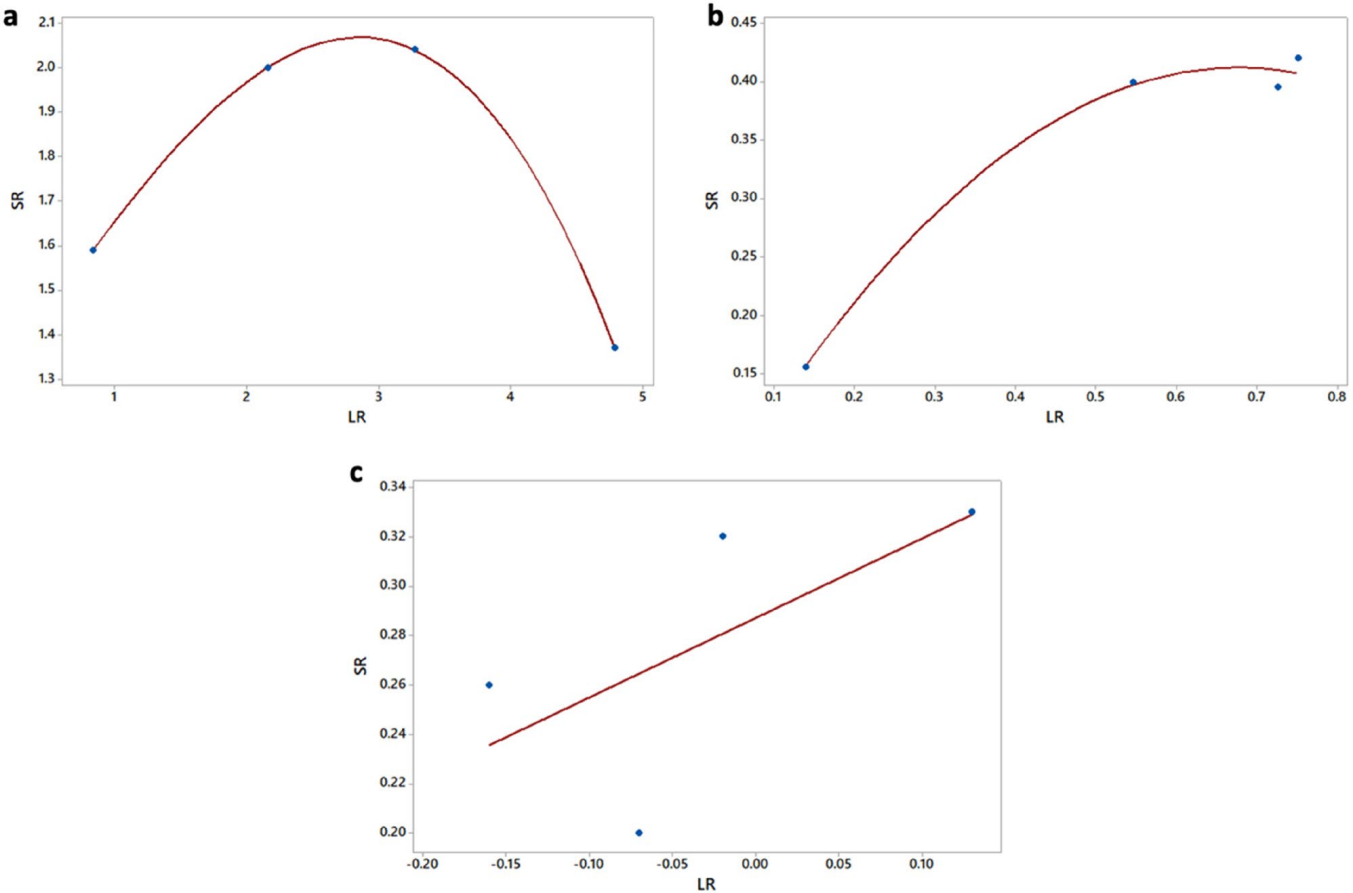
Reproducibility curves. Reproducibility SD (S_R_) calculated as a function of the mean log reduction (LR) for all 4 concentrations of NaOCl across all 5 labs. (**A**) Plate count, (**B**) Resazurin, (**C**) Crystal violet.

The reproducibility for all three methods is further illustrated in Fig. 3. In these graphs the reproducibility SD was plotted as a frown shaped function of the mean LR (across all 5 labs) for each NaOCl concentration. This is in contrast to the reproducibility SD presented in Table 3 that was calculated by pooling the data over all concentrations of NaOCl. For the plate count method (Fig. 3A), a similar relationship has been found for other antimicrobial test methods and micoorganisms^26^. Figures 3B and C show, for the first time in the literature, that the reproducibility SDs for resazurin and crystal violet appear to have this same frown shaped pattern. This suggests that microbial LRs, whether from CFUs, resazurin or crystal violet, are most reproducible for ineffective and highly effective antimicrobials.

To provide a comparison between the three methods that accounts for both responsiveness and reproducibility, we divided the slope of the dose–response curve by the reproducibility S_R_ (Slope/S_R_), analogous to the signal to noise ratio (and inversely related to the relative standard error and the limit of quantitation)^28^. The higher this unitless value the better the method is at evaluating the effect of different concentrations of treatment. The plate count method performs best, even though it had the worst reproducibility SD, because of the large change in LRs as the concentration of NaOCl was changed (i.e., because of the steep slope). The crystal violet method performs the worst in spite of having the best reproducibility because of poor responsiveness; thus, in our setup it is not suitable for differentiating antimicrobials with different efficacy. A potential reason for this poor responsiveness may be a peculiar interaction noticed between the bleach, the plastic material of the microtiter plate and the crystal violet dye (Fig. S4.7). In follow up experiments, when bleach at different NaOCl concentrations (2% vol/vol and 0.1% vol/vol) was added to an empty microtiter plate for 10 min and the plate was subsequently stained with 0.1% vol/vol crystal violet, the stain bound to the wells, despite the lack of biofilm. One reason for this could be that the bleach is corroding the surface of the wells and creates a rougher surface where the crystal violet can bind. Another possible explanation could be due to the redox reaction between crystal violet and bleach, which results in crystal violet losing colour. If any bleach is left behind following neutralisation then it might be affecting the crystal violet dye. Hence, when choosing a suitable method, it is important to take into consideration any chemical interactions between the antimicrobial agent, the material used to grow the biofilm and any staining agents used in the assessment method.

Moreover, from the dose–response curves for the plate count method in Fig. S4.4 it can be seen that for lab 3 the differences between the 4 NaOCl concentrations are not as pronounced as for the other labs. One reason for this difference in response was thought to be the relationship between the starting LD of the biofilm and the resulting LR, as biofilms with a lower starting LD are overall easier to kill by antimicrobial agents^29^. This can be seen in the graphs in Fig. S4.8, where the Mean LR for each lab was plotted as a function of the mean control LD. All 4 graphs show that for labs with higher mean control LDs, the mean LR values are lower. Hence, since lab 3 has overall lower control LD values (Fig. 1), the biofilm might be easier to kill with lower concentrations of NaOCl and saturation is reached, leading to no difference in LR between different treatments.

## Discussion

Despite the extensive use of microtiter plate methods in biofilm research, little is known about their comparability, limitations, repeatability, and reproducibility. Due to this lack of information data collected from microtiter plate methods often have little use outside of an individual study, or outside studies performed in the same laboratory. With this interlaboratory study we aimed to answer some of these questions: Is it possible to compare microtiter plate data across different labs? How repeatable and reproducible are crystal violet, resazurin and plate counts in microtiter plate experiments? How does the change in protocol for the assessment step affect reproducibility? How responsive and reproducible are these methods when performing an antimicrobial efficacy test?

First, our results clearly show that comparability between different labs is achievable. For the plate count method, this is very straightforward, however for resazurin and crystal violet extra steps and data transformations are necessary. Due to differences in plate reader outputs, to compare resazurin and crystal violet data across labs, it was imperative that each lab calculate their own calibration curves. An attempt was made to compare “raw” data without the use of calibration curves, and the repeatability and reproducibility were adversely affected [raw data available in Zenodo repository, https://doi.org/10.5281/zenodo.4450073]. The implementation of calibration curves enabled us to report the data as log_10_ concentration of crystal violet and resorufin, as opposed to the more commonly used optical density (OD) and fluorescence emission data. Use of calibration curves is not common practice for these methods and as such it is not possible to compare any published data from different research groups on these methods even if their protocols are similar or exactly the same. Hence, the way in which the scientific community treats and reports such data needs to change.

Once we made the data comparable, we examined how repeatable and reproducible each of these three assessment methods were in microtiter plates. If we focus solely on our study, the control experiment results showed that the crystal violet method is the most repeatable and reproducible method of the three, while plate counts were the least. However, previous studies on the repeatability of microtiter plate methods seem to be in contradiction with our results. In the study by Jorge et al. 2015, they evaluated the repeatability of the crystal violet, plate counts and XTT methods in assessing *Pseudomonas aeruginosa* biofilms. Their results showed issues with repeatability for all three methods, most noticeably for the crystal violet method^7^. Similarly, in the study by Kragh et al. 2019 they report better repeatability for the plate count method when compared to crystal violet for 24 h biofilms of *P. aeruginosa*^8^. The most probable explanation for these differences could be the way the data were treated. These studies do not use calibration curves for the crystal violet data and instead report them as OD values and they also do not log transform their plate count data. These observed differences in repeatability could also be due to differences in the bacterial species used, as our study was performed on *S. aureus* biofilms, not *P. aeruginosa*. Moreover, they could also be attributable to differences in the biofilm growth protocol and assessment methods protocols.

As the plate count method is extensively used in previously validated standard methods, we can compare the reproducibility of our ILP protocol to that observed for other biofilm growing devices^17,18,22^. For example, the MBEC device which has been accepted as a standard method for antimicrobial testing on *P. aeruginosa* biofilms^20^ is a modified microtiter plate where the biofilm grows on pegs attached to the microtiter plate lid. As such the environment and growth conditions within the MBEC device are similar to the microtiter plate. For the MBEC device the ring trial results showed a control S_R_ = 0.67 and Mean Log_10_ CFU/mm^2^ = 5.48^17^. If we convert our data to Log_10_ CFU/ mm^2^, the Mean Log_10_ CFU/mm^2^ = 5.14 and the control S_R_ remains the same at 0.92. Hence, our control data are not as reproducible as the MBEC device (Table 1). Nevertheless, acceptable repeatability and reproducibility SDs for plate counts from controls are < 0.5 and < 0.7, respectively^30^. In our case, the plate count method has very repeatable control data with an SD = 0.27, but less reproducible control data with an S_R_ = 0.92. No references are available for acceptable SD values for control data from the crystal violet and resazurin methods.

To compare the reproducibility of the LRs between microtiter plates and the MBEC, we can compare the reproducibility curve in Fig. 3A to the similar frown shaped curve showing reproducibility for the MBEC^26,29^. The reproducibility SD curve in Fig. 3A is higher than for the MBEC over the same range of LRs, peaking at about S_R_ = 2 for the microtiter method compared to S_R_ = 1.5 for the MBEC. This indicates that LRs generated from CFUs from the 96-well microtiter plate method are more variable than the MBEC when testing antimicrobials with a wide range of efficacy. One possible explanation for this could be the difference in the biofilm harvesting protocol. While the MBEC device relies on a mostly automated process by using sonication to remove the biofilm from the pegs, for the microtiter plate the biofilm is removed by scrapping each well individually, which may increase the chance for human error. Moreover, as the ILP protocol did not go through the ruggedness testing that the MBEC device and other standard methods went through, further optimisation could help improve the reproducibility of the method^17,31^. Nonetheless, as these tests were performed for different species of bacteria, it is not certain if this difference in reproducibility is due to the different bacteria or the protocols used or the device itself.

Another question we wanted to explore was whether using a standardised protocol for the assessment method made a difference in results. For this purpose, groups with established in-house protocols (IHP) performed them in parallel to our ILP. For all three methods, the results showed that the mean response was statistically significantly equivalent between the different protocols but that the reproducibility for the ILP was better (i.e., the S_R_ values for the ILP were smaller). One interesting detail to consider is that the biofilm growth protocol and the inoculum used was the same for both the ILP and the IHP protocols. This suggests that what contributes the most to variability is how the biofilm is grown and how the inoculum is prepared, rather than how the assessment of the biofilm is performed, an observation that is in agreement with previous research^32^.

Finally, we wanted to evaluate which assessment method would be the most appropriate to use for a treatment efficacy test. For this purpose, we chose NaOCl as the antimicrobial treatment and we evaluated NaOCl contact killing at four different NaOCl concentrations. An initial look at the results seemed to suggest that once again the crystal violet method was the most reproducible and as such the better method to use. However, when performing treatment efficacy testing, the responsiveness of the method is crucial. Responsiveness indicates the ability of the method to differentiate between different concentrations of the treatment and evaluate it as a dose–response. The plate count method was the most responsive method of the three. Furthermore, when we looked at their reproducibility with respect to responsiveness, the plate count method performed the best out of the three. Unexpectedly, the resazurin method which measures the metabolic activity of the biofilm showed poor responsiveness compared to plate counts. This could be due to a high presence of viable but dormant cells in the treated wells or an unknown interaction of resazurin with the antimicrobial used. Another possibility is that the observed responsiveness of the plate counting method is artificially too high due to a large number of viable but non-culturable survivors on treated carriers.

In conclusion, the microtiter plate is a versatile and easy-to-use biofilm reactor which shows good repeatability and reproducibility for different types of assessment methods. It can also allow for comparison of data between different labs. However, for comparisons across labs to be possible calibration curves are essential, hence there needs to be a change in the way we report microtiter plate experiment data, especially for spectrophotometric and fluorometric type assays. This combined with more detailed reporting based on minimum information guidelines could add more value to results obtained from microtiter plate experiments and open the possibility for comparison across studies and data mining applications.

## Supplementary Information

Supplementary Information.

## Data Availability

The datasets generated and analysed during the current study are available in the Zenodo repository, https://doi.org/10.5281/zenodo.4450073.

## Abbreviations

Mixed effects ANOVA: Statistical process for separating the variability of a group of observations into assignable sources and performing significance tests.
Error: Represents variability attributable to well-to-well differences.
Day: Represents variability attributable to day-to-day differences e.g., different inoculum, temperature, humidity, etc.
Lab: Represents variability attributable to lab-to-lab differences e.g., different technicians, equipment, climate, etc.
Repeatability: Represents the variability within each lab (error + day).
Reproducibility: Represents the variability among labs (total = error + day + lab).
Responsiveness: Sensitivity of a method; ability of a method to detect important changes in an antimicrobial’s efficacy (e.g., due to changes in concentration or contact time)

## Code availability

Minitab® and all other trademarks and logos for the Company's products and services are the exclusive property of Minitab, LLC. All other marks referenced remain the property of their respective owners. See minitab.com for more information. License Server: @software.fe.up.pt.
