# Supplementary material for: Interlaboratory study for the evaluation of three microtiter plate-based biofilm quantification methods
Source: Sci Rep. 2021 Jul 2;11:13779. doi: 10.1038/s41598-021-93115-w (PMC8253847; doi:10.1038/s41598-021-93115-w)
Supplement: Supplementary file 1 — Supplementary Information. [file 41598_2021_93115_MOESM1_ESM.docx]

**Interlaboratory study for the evaluation of three microtiter plate-based biofilm quantification methods**

Jontana Allkja^1,2^, Frits van Charante^3^, Juliana Aizawa^4^, Inés Reigada^5^, Clara Guarch-Pérez ^6^, Jesus Augusto Vazquez-Rodriguez^7^, Paul Cos^4^, Tom Coenye^3,8^, Adyary Fallarero^5^, Sebastian A.J. Zaat^6^, Antonio Felici^7^, Livia Ferrari^7^, Nuno F. Azevedo^1^, Albert E. Parker^2,9^, Darla M. Goeres^2,*^

*Corresponding author: E-mail: darla_g@montana.edu; Phone number: +1 406-994-2440

Author affiliations:

^1^ LEPABE - Laboratory for Process Engineering, Environment, Biotechnology and Energy, Faculty of Engineering, University of Porto, Rua Dr. Roberto Frias, 4200-465 Porto, Portugal

^2^ Montana State University, Center for Biofilm Engineering, 366 Barnard Hall, Bozeman, MT 59717, United States of America

^3^ Laboratory of Pharmaceutical Microbiology, Ghent University, Ghent, Belgium

^4^ Laboratory for Microbiology, Parasitology and Hygiene (LMPH), Faculty of Pharmaceutical, Biomedical and Veterinary Sciences, University of Antwerp, Antwerp, Belgium

^5^ Drug Research Program, Division of Pharmaceutical Biosciences, Faculty of Pharmacy, University of Helsinki, 00790 Helsinki, Finland

^6^ Department of Medical Microbiology and Infection Prevention, Amsterdam institute for Infection and Immunity, Amsterdam UMC, University of Amsterdam, 1105 AZ Amsterdam, The Netherlands

^7^ Discovery Microbiology, Aptuit (Verona) S.r.l., an Evotec Company, Verona, Italy

^8^ ESCMID Study Group for Biofilms, Basel, Switzerland

^9^ Department of Mathematical Sciences, Montana State University, Bozeman, MT, United States of America

**S1. Collection of protocols used in interlaboratory study**

Detailed description of the ILP protocols developed and used in the ring trial study, as well as in-house protocols from participating laboratories.

# Inter-lab Protocols

**Prior to performing the experiments for the interlaboratory study, conduct the following tests:**

1. Crystal violet: Make 0.001% crystal violet in laboratory grade (deionized water) and serially dilute by a factor of 2. Add 200 µL/well of each dilution to a microtiter plate using the following layout:

|  | 1 | 2 | 3 | 4 | 5 | 6 | 7 | 8 | 9 | 10 | 11 | 12 |
| --- | --- | --- | --- | --- | --- | --- | --- | --- | --- | --- | --- | --- |
| A |  |  |  |  |  |  |  |  |  |  |  |  |
| B |  |  |  |  |  |  |  |  |  |  |  |  |
| C |  |  |  |  |  |  |  |  |  |  |  |  |
| D |  |  |  |  |  |  |  |  |  |  |  |  |
| E |  |  |  |  |  |  |  |  |  |  |  |  |
| F |  |  |  |  |  |  |  |  |  |  |  |  |
| G |  |  |  |  |  |  |  |  |  |  |  |  |
| H |  |  |  |  |  |  |  |  |  |  |  |  |

|  | 0.001% Crystal violet |
| --- | --- |
|  | 0.001% Crystal violet / 2 |
|  | 0.001% Crystal violet / 4 |
|  | 0.001% Crystal violet / 8 |
|  | 0.001% Crystal violet / 16 |
|  | 0.001% Crystal violet / 32 |
|  | 0.001% Crystal violet / 64 |
|  | 0.001% Crystal violet / 128 |

Read the plate at 595 nm and record the data in the spreadsheet provided.

1. Resorufin: Make 5 µg/mL resorufin solution. Resorufin can be made by reducing resazurin using sodium dithionite (also known as sodium hydrosulfite) [Sigma Aldrich; #7775-14-6] or by using commercially available resorufin powder [Sigma Aldrich; #34994-50-8].

To reduce resazurin we suggest making a stock of 1 g/L resazurin in MilliQ water and adding sodium dithionite one grain at a time until the solution turn dark pink or red (Using stocks of lower concentrations will increase chances of over-reducing the resazurin until it becomes colorless). To keep this compound stable it should be prepared in anaerobic conditions and autoclaved.

Serially dilute by a factor of 2 in MilliQ water and add 200 µL/well of each dilution to a microtiter plate using the following layout:

|  | 1 | 2 | 3 | 4 | 5 | 6 | 7 | 8 | 9 | 10 | 11 | 12 |
| --- | --- | --- | --- | --- | --- | --- | --- | --- | --- | --- | --- | --- |
| A |  |  |  |  |  |  |  |  |  |  |  |  |
| B |  |  |  |  |  |  |  |  |  |  |  |  |
| C |  |  |  |  |  |  |  |  |  |  |  |  |
| D |  |  |  |  |  |  |  |  |  |  |  |  |
| E |  |  |  |  |  |  |  |  |  |  |  |  |
| F |  |  |  |  |  |  |  |  |  |  |  |  |
| G |  |  |  |  |  |  |  |  |  |  |  |  |
| H |  |  |  |  |  |  |  |  |  |  |  |  |

|  | 5 µg/mL resorufin |
| --- | --- |
|  | 2.5 µg/mL resorufin |
|  | 1.25 µg/mL resorufin |
|  | 0.625 µg/mL resorufin |
|  | 0.313 µg/mL resorufin |
|  | 0.156 µg/mL resorufin |
|  | Water |

Measure fluorescence at λ_excitation_ = 560 nm; λ_emission_ = 590 nm and record the data in the spreadsheet provided.

**Experimental design**

Each laboratory needs to perform 3 different biofilm assessment protocols: crystal violet, resazurin and CFU count. All experiments must be performed by only one person per lab. For each protocol 2 different plate layouts will be tested. A control plate to assess the variability within the plate and a treatment plate to be used for a disinfectant activity test, to assess the sensitivity of the methods. Each protocol lasts 4 days, expect for the CFU count which lasts for 5 days. The first three days are the biofilm formation part of the protocol and as such they will be the same for all methods. When performing the control plate experiments, we ask that each laboratory uses their own protocol (IHP) on the 4^th^ day (data collection day) in parallel with the inter-lab protocol (ILP), if available. Repeat the experiments twice for the control plates and 3 times for the treatment plates. Below you can find a suggested timeline for the ring trial. In total all experiments should take 6 weeks to be completed.

| **Monday** | **Tuesday** | **Wednesday** | **Thursday** | **Friday** |
| --- | --- | --- | --- | --- |
| Day 1 | Day 2 | Day 3 | Data collection day |  |
|  | Day 1 | Day 2 | Day 3 | Data collection day |

**Table 1.** Model week layout

**Table 2.** Suggested ring trial timeline

| **Data collection day 1** | **Data collection day 2** | **Data collection day 3** | **Data collection day 4** | **Data collection day 5** | **Data collection day 6** | **Data collection day 7** | **Data collection day 8** | **Data collection day 9** | **Data collection day 10** | **Data collection day 11** | **Data collection day 12** |
| --- | --- | --- | --- | --- | --- | --- | --- | --- | --- | --- | --- |
| Control plate Crystal violet ILP + IHP | Control plate Resazurin ILP + IHP | Control plate CFU count ILP + IHP | Control plate Crystal violet ILP + IHP | Control plate CFU count ILP + IHP | Control plate Resazurin ILP + IHP | Treated plate CFU count + Crystal violet | Treated plate Resazurin | Treated plate CFU count + Crystal violet | Treated plate Resazurin | Treated plate CFU count + Crystal violet | Treated plate Resazurin |

**Disinfectant preparation protocol**

Sodium hypochlorite (NaOCl) or bleach will be used to challenge the biofilm. The disinfectant will be diluted to 1000 mg/L fresh every day, no more than 2 hours prior to use. Additionally, a titration test needs to be performed to make sure the total concentration of chlorine within your solution is 1000 ±10 mg/L. To perform the titration test you can use a digital titrator [Hach; Mfr. No. 1690001] or any commercially available total chlorine test that falls within this range.

**Dilution**

Bleach [Pure Bright] stock concentration 6 % i.e. 60000 mg/L. Calculate amount of stock needed to make 30 mL of 1000 mg/L solution using C_1_V_1_=C_2_V_2_ formula. Dilute 0.5 mL of stock bleach solution into 29.5 mL of filter sterilized MilliQ water. **Make sure the container used is sterile and try to avoid plastic containers.** Perform a titration test to confirm the solution is within the 10 % range as described above.

Perform the following dilutions:

- 5 mL of 1000 mg/L + 5 mL of sterile MilliQ water = 500 mg/L
- 1 mL of 1000 mg/L + 9 mL of sterile MilliQ water = 100 mg/L
- 0.1 mL of 1000 mg/L + 9.9 mL of sterile MilliQ water = 10 mg/L
- Keep an extra 15 mL of sterile MilliQ water to use for the experiment


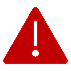


**Make sure to adjust the calculations depending on the concentration stated on the label of your bleach bottle and perform the titration test every day.**

**Biofilm growth Protocol**

**Day 1**

Materials: Tryptic Soy Agar (TSA) [Difco, Cat #236290] plate.

*Staphylococcus aureus subsp. aureus* (ATCC® 25923™) glycerol stock, stored at -80 °C.

Protocol: Using a sterile inoculation loop pick up some of the *Staphylococcus aureus subsp. aureus* (ATCC® 25923™) glycerol stock and streak it on a TSA plate. Incubate the plate at 37 ± 2 °C for 24 hours.

**
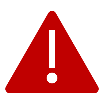
If possible use stocks only once per experiment. Alternatively, if stocks are re-used make sure that no contamination is present and colony morphology is consistent.**


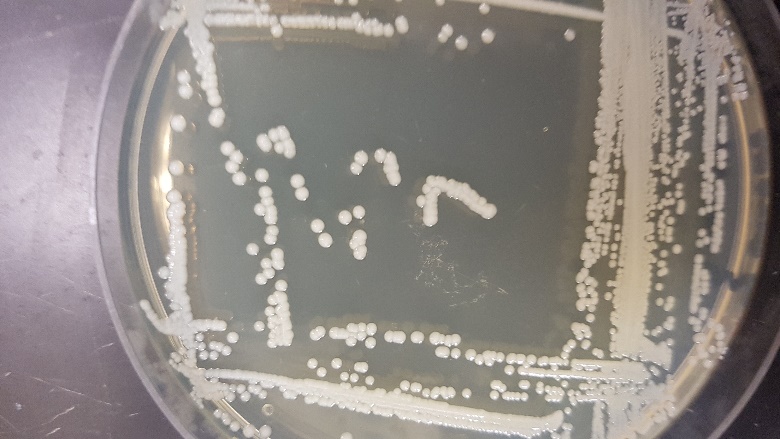
The colonies should be round, smooth, slightly raised and light yellow/creamy white in colour. Typical colony appearance and size is shown in Figure 1. If colonies formed are too small, too large, or drastically different in appearance and morphology, use fresh stock.

**Figure 1.** *Staphylococcus aureus subsp. aureus* (ATCC® 25923™) colony morphology

**Day 2**

Materials and equipment: Tryptic Soy Broth (TSB) [Difco, Cat # 211822] conc. 30 g/L.

Sterile 50 mL Falcon tube

Orbital shaker

Protocol: Prefill falcon tube with 15 mL of TSB.

Using a sterile inoculation loop pick one colony from the day 1 streak plate and transfer it into the falcon tube. Incubate the inoculum at 37 ± 2 °C, 125 rpm, 1.9 cm orbital diameter for 18 hours. (Suggested time to incubate 15:00)

**Day 3**

Materials and Equipment: Tryptic Soy Broth (TSB) [Difco, Cat # 211822] conc. 30 g/L.

Sterile 50 mL Falcon tube

Thermo Scientific Nunc Flat bottom sterile 96 well plate (Cat # 12-566-202)

Dilution tubes

Spectrophotometer

Vortexer

Orbital shaker

Protocol: Prefill 14 dilution tubes with 9 mL of TSB and 1 falcon tube with 9.9 mL TSB.

Remove the day 2 inoculum from incubation and vortex to mix. Pipette 100 µL of the inoculum into 9.9 mL of TSB. Incubate at 37 ± 2 °C, 125 rpm, 1.9 cm orbital diameter for approx. 3 hours or until optical density at 595 nm reaches 0.300 ± 0.020 (Use TSB as blank). Dilute the new inoculum 1:100 (total volume/plate = 20 mL) in fresh TSB and vortex to mix. Pipette 200 µL/well of the 1:100 dilution into two microtiter plates, following layout 1 for the control plate. Alternatively, for the treatment plate follow layouts 2 or 3 (depending on the assessment method to be performed) and only prepare 1 microtiter plate.

|  | **1** | **2** | **3** | **4** | **5** | **6** | **7** | **8** | **9** | **10** | **11** | **12** |
| --- | --- | --- | --- | --- | --- | --- | --- | --- | --- | --- | --- | --- |
| **A** |  |  |  |  |  |  |  |  |  |  |  |  |
| **B** |  |  |  |  |  |  |  |  |  |  |  |  |
| **C** |  |  |  |  |  |  |  |  |  |  |  |  |
| **D** |  |  |  |  |  |  |  |  |  |  |  |  |
| **E** |  |  |  |  |  |  |  |  |  |  |  |  |
| **F** |  |  |  |  |  |  |  |  |  |  |  |  |
| **G** |  |  |  |  |  |  |  |  |  |  |  |  |
| **H** |  |  |  |  |  |  |  |  |  |  |  |  |

|  | TSB only |
| --- | --- |
|  | *S. aureus* only |

**Layout 1**

|  | CFU count | | | | | | Crystal violet | | | | | |
| --- | --- | --- | --- | --- | --- | --- | --- | --- | --- | --- | --- | --- |
|  | **1** | **2** | **3** | **4** | **5** | **6** | **7** | **8** | **9** | **10** | **11** | **12** |
| **A** |  |  |  |  |  |  |  |  |  |  |  |  |
| **B** |  |  |  |  |  |  |  |  |  |  |  |  |
| **C** |  |  |  |  |  |  |  |  |  |  |  |  |
| **D** |  |  |  |  |  |  |  |  |  |  |  |  |
| **E** |  |  |  |  |  |  |  |  |  |  |  |  |
| **F** |  |  |  |  |  |  |  |  |  |  |  |  |
| **G** |  |  |  |  |  |  |  |  |  |  |  |  |
| **H** |  |  |  |  |  |  |  |  |  |  |  |  |
|  |  |  |  |  |  |  |  |  |  |  |  |  |
|  |  |  |  |  |  |  |  |  |  |  |  |  |

**Layout 2**

|  | Resazurin | | | | | | Water | | | | | |
| --- | --- | --- | --- | --- | --- | --- | --- | --- | --- | --- | --- | --- |
|  | **1** | **2** | **3** | **4** | **5** | **6** | **7** | **8** | **9** | **10** | **11** | **12** |
| **A** |  |  |  |  |  |  |  |  |  |  |  |  |
| **B** |  |  |  |  |  |  |  |  |  |  |  |  |
| **C** |  |  |  |  |  |  |  |  |  |  |  |  |
| **D** |  |  |  |  |  |  |  |  |  |  |  |  |
| **E** |  |  |  |  |  |  |  |  |  |  |  |  |
| **F** |  |  |  |  |  |  |  |  |  |  |  |  |
| **G** |  |  |  |  |  |  |  |  |  |  |  |  |
| **H** |  |  |  |  |  |  |  |  |  |  |  |  |
|  |  |  |  |  |  |  |  |  |  |  |  |  |
|  |  |  |  |  |  |  |  |  |  |  |  |  |

|  | TSB only |
| --- | --- |
|  | *S. aureus* only |
|  | Water |

**Layout 3**


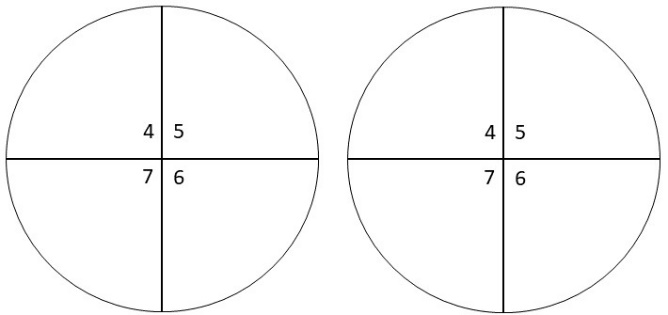
Serially dilute both inoculums by a factor of 10 up to 10^-7^, and drop plate (10 µL/drop), 5 drops/ concentration, 2 plates per concentration as shown on TSA plates below:


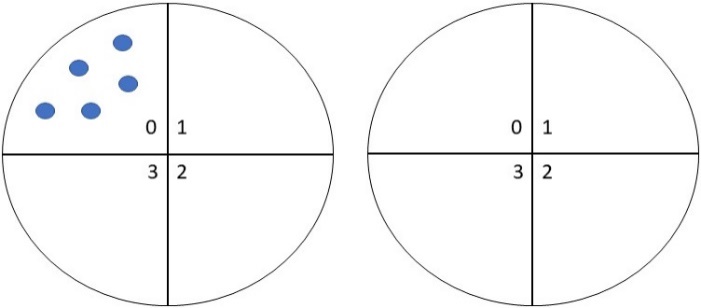
 Incubate TSA plates at 37 ± 2 °C for 24 hours and count colonies the next day. Input the colony count numbers in the spreadsheet provided. Resulting CFUs for the OD592 = 0.300 inoculums should be in the range of 7.5 ± 0.5 Log CFU/mL and the 1:100 dilutions should be in the range of 5.5 ± 0.5 Log CFU/mL.

For the control plates: Incubate one microtiter plate following your own laboratory’s protocol. For the second microtiter plate, parafilm the sides and incubate at 37 ± 2 °C, no shaking for 24 hours.

For the treatment plates: Parafilm the sides of the plate and incubate at 37 ± 2 °C, no shaking for 24 hours.

If you notice condensation on your plates or if the climate in your location is very dry, put a tray of water at the bottom of the incubator.

**Day 4 (Data collection day) Control plate**

For microtiter plate 1 if applicable use your own laboratory’s protocol and run parallel to the corresponding ILP protocol. Run the protocols according to the plan in table 2.

**CFU count ILP**

Materials: Thermo Scientific Nunc Flat bottom sterile 96 well plates (Cat # 12-566-202)

Tryptic Soy Broth (TSB) [Difco, Cat # 211822] conc. 30 g/L.

Sterile wooden applicator sticks [Fisher brand, Cat # 01-340]

Phosphate Buffered Saline (PBS) [pH 7.4, 8 g/L NaCl, 0.2 g/L KCl, 1.44 g/L Na_2_HPO_4_ and 0.24 g/L KH_2_PO_4_]

Protocol: Prefill 96 well plates with fresh TSB (yellow wells), 180 µL/well according to the following layout:

|  | 1 | 2 | 3 | 4 | 5 | 6 | 7 | 8 | 9 | 10 | 11 | 12 |
| --- | --- | --- | --- | --- | --- | --- | --- | --- | --- | --- | --- | --- |
| A |  |  |  |  |  |  |  |  |  |  |  |  |
| B |  |  |  |  |  |  |  |  |  |  |  |  |
| C |  |  |  |  |  |  |  |  |  |  |  |  |
| D |  |  |  |  |  |  |  |  |  |  |  |  |
| E |  |  |  |  |  |  |  |  |  |  |  |  |
| F |  |  |  |  |  |  |  |  |  |  |  |  |
| G |  |  |  |  |  |  |  |  |  |  |  |  |
| H |  |  |  |  |  |  |  |  |  |  |  |  |

|  | 180 µL TSB |
| --- | --- |

Remove planktonic suspension from the microtiter plate by pipetting. Do not tilt plate and insert the pipette tip at a 45° angle making sure not to touch the sides or the bottom of the well. **(If any contaminated control wells are present discard the plate!)**

Wash the plate twice with 250 µL/well of PBS. Pipette the PBS in and out making sure not to disturb the biofilm. Leave the plates to dry for 10-15 mins (lid off, in laminar flow).

Add 200 µL/well of fresh TSB to each well. Use a wooden applicator stick to scrape the biofilm. Scrape the sides of the well and the bottom (3x each), shake the stick in the well after scraping to remove some of the biofilm left behind on the stick. **(Video guide available at:** [**Training video**](https://drive.google.com/file/d/1iwjbBw5oIbHQMcEIGz71uMLgw2eLeqgG/view?usp=sharing)**)**

Scrape 15 wells/plate using the layouts below:

| **Pattern 1** | | |  |  |  |  |  |  |  |  |  |  |
| --- | --- | --- | --- | --- | --- | --- | --- | --- | --- | --- | --- | --- |
|  | **1** | **2** | **3** | **4** | **5** | **6** | **7** | **8** | **9** | **10** | **11** | **12** |
| **A** |  |  |  |  |  |  |  |  |  |  |  |  |
| **B** |  |  |  |  |  |  |  |  |  |  |  |  |
| **C** |  |  |  |  |  |  |  |  |  |  |  |  |
| **D** |  |  |  |  |  |  |  |  |  |  |  |  |
| **E** |  |  |  |  |  |  |  |  |  |  |  |  |
| **F** |  |  |  |  |  |  |  |  |  |  |  |  |
| **G** |  |  |  |  |  |  |  |  |  |  |  |  |
| **H** |  |  |  |  |  |  |  |  |  |  |  |  |
|  |  |  |  |  |  |  |  |  |  |  |  |  |
| **Pattern 2** | | |  |  |  |  |  |  |  |  |  |  |
|  | **1** | **2** | **3** | **4** | **5** | **6** | **7** | **8** | **9** | **10** | **11** | **12** |
| **A** |  |  |  |  |  |  |  |  |  |  |  |  |
| **B** |  |  |  |  |  |  |  |  |  |  |  |  |
| **C** |  |  |  |  |  |  |  |  |  |  |  |  |
| **D** |  |  |  |  |  |  |  |  |  |  |  |  |
| **E** |  |  |  |  |  |  |  |  |  |  |  |  |
| **F** |  |  |  |  |  |  |  |  |  |  |  |  |
| **G** |  |  |  |  |  |  |  |  |  |  |  |  |
| **H** |  |  |  |  |  |  |  |  |  |  |  |  |


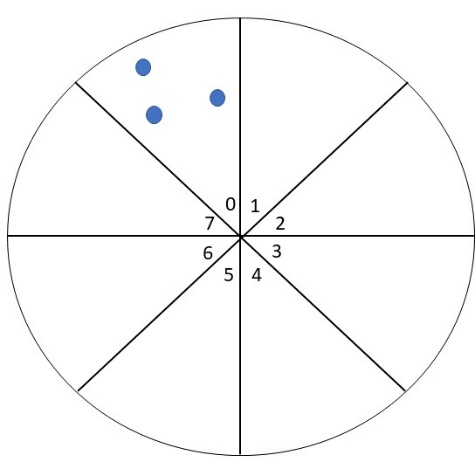
Mix the samples by pipetting and transfer to the top row of the prefilled plate. Serially dilute each sample by a factor of 10 going from row A (10^0^) to row H (10^-7^). Drop plate (10 µL/drop) on TSA plates using the following layout:

Incubate the plates at 37 ± 2 °C, for 24 hours. Count colonies the next day and input values in the spreadsheet provided.

**Crystal violet ILP**

Materials and equipment: PBS

3.5 g/L (vol/vol) Aqueous Crystal violet [Millipore Sigma; Cat #65092A-95]

100 % Ethanol

Sterile water

Plate reader

Protocol: Remove planktonic suspension from the microtiter plate by pipetting. Do not tilt plate and insert the pipette tip at a 45° angle making sure not to touch the sides or the bottom of the well. **(If any contaminated control wells are present discard the plate!)**

Wash the plate twice with 250 µL/well of PBS. Pipette the PBS in and out making sure not to disturb the biofilm. Leave the plates to dry for 10-15 mins (lid off, in laminar flow).

Add 200 µL/well of 99-100 % Ethanol to the plate and incubate for 15 mins at room temperature (20 ± 5 °C), no shaking. Then remove the ethanol by pipetting. (Do not tilt plate and insert the pipette tip at a 45° angle making sure not to touch the sides or the bottom of the well.)

Leave the plates to **dry fully** (lid off, in laminar flow) until no more ethanol is present in the wells.

Add 200 µL/well of 0.1 % (vol/vol) Crystal violet solution (diluted from stock in deionized water) and incubate at room temperature (20 ± 5 °C), no shaking for 15 min. Then remove the stain by pipetting. (Do not tilt plate and insert the pipette tip at a 45° angle making sure not to touch the sides or the bottom of the well.)

**
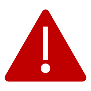
 Alternative Crystal violet stocks may be used. Make sure they are diluted to 0.1 % in deionized water!**

Wash the plate twice with 250 µL/well of sterile water. Pipette the water in and out making sure not to disturb the biofilm. **(Make sure no drops of crystal violet are left behind on the sides or rims of the wells!)** Leave the plates to dry for 10-15 mins (lid off, in laminar flow).

Add 200 µL/well of 99-100 % Ethanol to the plate and incubate for 30 mins at room temperature (20 ± 5 °C), 125 rpm. Read the absorbance at 595 nm and input raw data in the spreadsheet provided. If values are above 1.300 dilute in 99-100 % ethanol and provide dilution factor with the data.

**Resazurin ILP**

Materials and equipment: PBS

MilliQ water

Resazurin (stock 0.1 mg/mL) [BTC; Cat #214215-1G]

Protocol: Remove planktonic suspension from the microtiter plate by pipetting. Do not tilt plate and insert the pipette tip at a 45° angle making sure not to touch the sides or the bottom of the well. **(If any contaminated control wells are present discard the plate!)**

Wash the plate twice with 250 µL/well of PBS. Pipette the PBS in and out making sure not to disturb the biofilm. Leave the plates to dry for 10-15 mins (lid off, in laminar flow).

Make a 1:20 dilution (working solution = 5 µg/mL) of Resazurin stock in MilliQ water or sterile PBS, under sterile conditions and keep away from light.

Add 200 µL/well of working solution to the microtiter plate. Cover the plate in foil and incubate at room temperature (20 ±5 °C) for 60-90 minutes **(Depending on lab conditions this might take less or more time. Monitor color change and adjust time accordingly!)**, 125 rpm.

Measure fluorescence λ_excitation_ = 560 nm; λ_emission_ = 590 nm. Input the raw data into the spreadsheet provided.


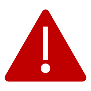
 **Other resazurin solutions can be used if the concentration of resazurin or the recommended fluorescence are the same as above.**

**Day 4 (Data collection day) Treatment plate**

**CFU count / Crystal violet**

Materials and equipment: PBS

3.5 g/L (vol/vol) Aqueous Crystal violet [Millipore Sigma; Cat #65092A-95]

100 % Ethanol

TSB

Wooden applicator sticks

Bleach

MilliQ water

Sterile water

Plate reader

Protocol: Prefill 96 well plates with 180 µL/well as on page 11.

Start on the crystal violet side of the plate (Columns 7 to 12).

Remove planktonic suspension from the microtiter plate by pipetting. Do not tilt plate and insert the pipette tip at a 45° angle making sure not to touch the sides or the bottom of the well. **(If any contaminated control wells are present discard the plate!)**

Wash the plate once with 250 µL/well of PBS. Pipette the PBS in and out making sure not to disturb the biofilm.

Repeat the process on the CFU count side (Columns 1-6).

Starting on the Crystal violet side add 250 µL/well of disinfectant following the layout below:

|  |  |  |  |  |  |  |  |  |  |  |  |  |
| --- | --- | --- | --- | --- | --- | --- | --- | --- | --- | --- | --- | --- |
|  | CFU count | | | | | | Crystal violet | | | | | |
|  | **1** | **2** | **3** | **4** | **5** | **6** | **7** | **8** | **9** | **10** | **11** | **12** |
| **A** |  |  |  |  |  |  |  |  |  |  |  |  |
| **B** |  |  |  |  |  |  |  |  |  |  |  |  |
| **C** |  |  |  |  |  |  |  |  |  |  |  |  |
| **D** |  |  |  |  |  |  |  |  |  |  |  |  |
| **E** |  |  |  |  |  |  |  |  |  |  |  |  |
| **F** |  |  |  |  |  |  |  |  |  |  |  |  |
| **G** |  |  |  |  |  |  |  |  |  |  |  |  |
| **H** |  |  |  |  |  |  |  |  |  |  |  |  |

|  | MilliQ |
| --- | --- |
|  | MilliQ |
|  | 1000 mg/L Chlorine |
|  | 500 mg/L Chlorine |
|  | 100 mg/L Chlorine |
|  | 10 mg/L Chlorine |

Use a multichannel pipette and add each solution 2 minutes apart. When exactly 10 minutes have passed remove the disinfectant and wash twice with 250 µL/well of PBS. **(If you find that 2-minute intervals are too short you can also increase it to 3 minutes apart.)**

Leave the crystal violet side of the plate to dry with the lid off while you repeat the process on the CFU count side. Once done add 200 µL/well of 99-100 % ethanol to columns 7-12 and incubate on the bench for 15 minutes. Then remove the ethanol by pipetting.

While the ethanol dries add 200 µL/well of TSB to the wells in column (1-6) following the patterns below:

|  | **Pattern 1** | | |  |  |  |
| --- | --- | --- | --- | --- | --- | --- |
|  | **1** | **2** | **3** | **4** | **5** | **6** |
| **A** |  |  |  |  |  |  |
| **B** |  |  |  |  |  |  |
| **C** |  |  |  |  |  |  |
| **D** |  |  |  |  |  |  |
| **E** |  |  |  |  |  |  |
| **F** |  |  |  |  |  |  |
| **G** |  |  |  |  |  |  |
| **H** |  |  |  |  |  |  |

|  | **Pattern 2** | | |  |  |  |
| --- | --- | --- | --- | --- | --- | --- |
|  | **1** | **2** | **3** | **4** | **5** | **6** |
| **A** |  |  |  |  |  |  |
| **B** |  |  |  |  |  |  |
| **C** |  |  |  |  |  |  |
| **D** |  |  |  |  |  |  |
| **E** |  |  |  |  |  |  |
| **F** |  |  |  |  |  |  |
| **G** |  |  |  |  |  |  |
| **H** |  |  |  |  |  |  |

|  | **Pattern 3** | | |  |  |  |
| --- | --- | --- | --- | --- | --- | --- |
|  | **1** | **2** | **3** | **4** | **5** | **6** |
| **A** |  |  |  |  |  |  |
| **B** |  |  |  |  |  |  |
| **C** |  |  |  |  |  |  |
| **D** |  |  |  |  |  |  |
| **E** |  |  |  |  |  |  |
| **F** |  |  |  |  |  |  |
| **G** |  |  |  |  |  |  |
| **H** |  |  |  |  |  |  |

|  | Wells to scrape |
| --- | --- |

Scrape the wells, serially dilute and drop plate using the same technique described on pages 11-12. Counts the plates the next day and record the data in the spreadsheet provided.

When done with the CFU counts, add 200 µL/well of 0.1 % (vol/vol) Crystal violet solution (diluted from stock in deionized water) and incubate at room temperature (20 ± 5 °C), no shaking for 15 min. Then remove the stain by pipetting. (Do not tilt plate and insert the pipette tip at a 45° angle making sure not to touch the sides or the bottom of the well.)

Wash the plate twice with 250 µL/well of sterile water. Pipette the water in and out making sure not to disturb the biofilm. **(Make sure no drops of crystal violet are left behind on the sides or rims of the wells!)** Leave the plates to dry for 10-15 mins (lid off, in laminar flow).

Add 200 µL/well of 99-100 % Ethanol to the entire plate and incubate for 30 mins at room temperature (20 ± 5 °C), 125 rpm. Read the absorbance at 595 nm and input raw data in the spreadsheet provided. If absorbance values are above 1.300 dilute in 99-100 % ethanol and provide dilution factor with the data.

**Resazurin**

Materials and equipment: PBS

MilliQ water

Bleach

Resazurin (stock 0.1 mg/mL) [BTC; Cat #214215-1G]

Plate reader

Protocol: Remove planktonic suspension from columns 1-6 of the microtiter plate by pipetting. Do not tilt plate and insert the pipette tip at a 45° angle making sure not to touch the sides or the bottom of the well. **(If any contaminated control wells are present discard the plate!)**

Wash the plate once with 250 µL/well of PBS. Pipette the PBS in and out making sure not to disturb the biofilm.

|  | MilliQ |
| --- | --- |
|  | MilliQ |
|  | 1000 mg/L Chlorine |
|  | 500 mg/L Chlorine |
|  | 100 mg/L Chlorine |
|  | 10 mg/L Chlorine |

Add 250 µL/well of disinfectant following the layout below:

|  | Resazurin | | | | | | Water | | | | | |
| --- | --- | --- | --- | --- | --- | --- | --- | --- | --- | --- | --- | --- |
|  | **1** | **2** | **3** | **4** | **5** | **6** | **7** | **8** | **9** | **10** | **11** | **12** |
| **A** |  |  |  |  |  |  |  |  |  |  |  |  |
| **B** |  |  |  |  |  |  |  |  |  |  |  |  |
| **C** |  |  |  |  |  |  |  |  |  |  |  |  |
| **D** |  |  |  |  |  |  |  |  |  |  |  |  |
| **E** |  |  |  |  |  |  |  |  |  |  |  |  |
| **F** |  |  |  |  |  |  |  |  |  |  |  |  |
| **G** |  |  |  |  |  |  |  |  |  |  |  |  |
| **H** |  |  |  |  |  |  |  |  |  |  |  |  |

Use a multichannel pipette and add each solution 2 minutes apart. When exactly 10 minutes have passed remove the disinfectant and wash twice with 250 µL/well of PBS. Leave to dry for 10-15 with the lid off under laminar flow. **(If you find that 2-minute intervals are too short you can also increase it to 3 minutes apart.)**

Make a 1:20 dilution (working solution = 5 µg/mL) of Resazurin stock in MilliQ water or sterile PBS, under sterile conditions and keep away from light.

Add 200 µL/well of working solution to the microtiter plate. Cover the plate in foil and incubate at room temperature (20 ±5 °C) for 1-2 hours **(Depending on lab conditions this might take less or more time. Monitor color change and adjust time accordingly!)**, 125 rpm.

Measure fluorescence λ_excitation_ = 560 nm; λ_emission_ = 590 nm. Input the raw data into the spreadsheet provided.

**Supply list**

| **Item** | **Supplier / Brand** | | **Concentration** | **Storage** | |  |
| --- | --- | --- | --- | --- | --- | --- |
| **Bleach** | Pure Bright | | 6 % NaOCl v/v | Shelf, Room temperature | |  |
| **Crystal violet** | Millipore Sigma; Cat #65092A-95 | | 3.5 g/L (0.35%) v/v | Shelf, Room temperature | |  |
| **Deionized water** | Laboratory water system | | N/A | N/A | |  |
| **Ethanol** | Decon™ Labs, Cat # 2705HC | | 200 Proof (100%) | Shelf, Room temperature | |  |
| **Flat bottom, sterile, polystyrene, 96 well plates** | Thermo Scientific, Cat # 12-566-202 | | N/A | Shelf, Room temperature | |  |
| **MilliQ water** | Laboratory water system | | N/A | N/A | |  |
| **Phosphate buffered Saline** | Made in-house  Sterilize by autoclaving or filtration | | 137 mM NaCl; 2.7 mM KCl;  10 mM Na_2_HPO_4_;  1.8 mM KH_2_PO_4_.  pH 7.4 | Shelf, Room temperature | |  |
| **Resazurin** | BTC; Cat #214215-1G | | Stocks made at 0.1 mg/mL in PBS | Powder (Shelf)  Stock (+ 4 °C; dark) | |  |
| **Sodium dithionite** | Sigma Aldrich; Cat #7775-14-6 | | N/A | Shelf | |  |
| ***Staphylococcus aureus subsp. aureus*** | ATCC® 25923™ | | Glycerol stocks | -80 °C | |  |
| **Syringe filters, Sterile** | Corning, Cat # 431224 | | 0.22 µm Nylon pore | Shelf | |  |
| **Tryptic Soy Agar** | Difco, Cat #236290 | | 40 g/L | Shelf | |  |
| **Tryptic Soy Broth** | Difco, Cat # 211822 | | 30 g/L | Shelf | |  |
| **Wooden applicator sticks** | | Fisher brand, Cat # 01-340 | N/A | | Drawer | |

# Protocols UGhent

***Crystal violet staining***

After growing a biofilm in a 96-well microtiter plate:

- - Remove the supernatant
  - Wash with 100 µL physiological saline
  - Add 100 µL 99% methanol to each well (denaturation and dehydration of the cells => fixation)
  - After 15 min, remove the methanol by turning the plate upside down above the special container in the sink
  - Dry plates in the incubator (37°C) until all methanol is evaporated (place microtiter plate without lid in an incubator)
  - Add 100 µL of a 0.1% crystal violet solution to each well (stock solution is 0.5%)
  - Let stand on the bench for 20 min
  - After 20 min, put on gloves and remove the crystal violet solution by turning the plate above the container in the sink
  - Wash under running tap water and dry plates by placing them on absorbent paper
  - Add 150 µL of a 33% acetic acid solution
  - Incubate the microtiter plate on a rotator (450 rpm) for at least 20 minutes (all crystal violet should be in solution)
  - Measure the absorbance of all wells at 590 nm ( EnVision protocol for round bottomed plates: KV abs @ 590 nm)

Comments:

It is not necessary to work sterile!

Average absorbances at 590 nm are calculated and the net absorbances are determined by taking into account the absorbance values of the negative growth controls (=blanc values).

***Resazurin staining***

After growing a biofilm in a 96-well microtiterplate:

- - Remove the supernatant
  - Wash with 100 µL physiological saline
  - Prepare a resazurin solution by adding 10.5 mL physiological saline to 2.1 mL aliqots of resazurin stored in the freezer (-20°C) (1/6 dilution of commercial available CTB solution Promega)
  - Add 120 µL resazurin solution to each well (first the blanks)
  - Incubate 1 hour (or more depending on the strain using) at 37°C (also strain dependent) **protected from light**

(time: 20min for *P. acnes,* 30min for *S. aureus,* 1h for *B. cenocepacia*)

- - Measure fluorescence (l_ex_: 560 nm and l_em_: 590 nm (Nvision: CTB Heleen) Calculate the mean of different wells and correct for blank fluorescence values

Work sterile!

**CFU count for biofilms from 96 well plate**

After growing/treating biofilm

- Remove supernatant
- Wash biofilms with 100 µL physiological saline
- Add 100 µL physiological saline to each well
- Replace the lid with a sealing film
- Vortex the 96 well plate for 5 min at 900 rpm
- Place the 96 well plate in the sonificator (42 Hz ± 6%) for 5 min
- Pipette the 100 µL out of the wells and add to 1.8 mL physiological saline
- Add 100 µL physiological saline to each well
- Vortex the 96 well plate for 5 min at 900 rpm
- Place the 96 well plate in the sonificator (42 Hz ± 6%) for 5 min
- Pipette the 100 µL out of the wells and add to the same 1.8 mL physiological saline
- Pipette 1 mL from the now 2 mL physiological saline (10^-1) and add to 9mL physiological saline (10^-2)
- Repeat until desired dilution(s)
- Pipette 1 mL of desired dilution(s) into petri dish
- Add 15-20 mL of agar to petri dish and mix to distribute cells (MHA for *S. aureus*)

# Protocols UMC, Amsterdam

***Culturing; 96 wells plate Biofilm dispersion by sonication***

- Sonicate the micro titer plate for 5 minutes in a ultrasonic water bath (Elma; Transsonic 460; 35 Hz):
  - Use a sealing film (Greiner; easy seal) to cover the micro titer plate (press carefully on each well), put a lid on the plate and seal it with parafilm. Put the micro titer plate in a plastic bag, which must also be sealed to prevent contamination during sonication. (This construction can float in the ultrasonic water bath.)
- After sonication pipet several times check visually (microscopically if necessary) if the biofilm has been dispersed properly.
- Take ~100 μl from the sonicated biofilm plate and add it to the first column of a fresh plate to make dilutions.
- Plate dilutions on blood agar plates (for example 2 x 10 µ).

***96 wells plate Biofilm detection using Crystal violet staining***

- Wash the biofilm very carefully 2x (or more depending on you assay) by pipetting off the supernatant (only in the corner with your tip) and adding PBS. Repeat once, second wash only remove the PBS.
- Stain adherent bacteria with 125 μl of 1% crystal violet for **10 min** at RT.
- Wash wells 4x with water and dry plate on paper towels.
- Add 150 μl 96% ethanol to each well to dissolve crystal violet. Incubate **10 min** at RT.
- Transfer 100 μl from each well to a new 96-wells flat bottom plate. Measure optical density at 595 nm.

# Protocols UHelsinki

***B1. Resazurin staining***

- Make a 1:20 dilution of the resazurin stock in PBS
- Wash the biofilms once with 200 µL PBS (or MQ-water), carefully to avoid harming the biofilms
- Add 200 µL of the resazurin diluted solution per well
- Incubate in RT, darkness, 200 rpm for about 45 min (for *S. aureus 25923* but *P. aeruginosa* needs about 1h30)
- Measure fluorescence at λ_excitation_ = 560nm and λ_emission_ = 590nm

***B2. Crystal violet staining***

- Remove the resazurin stain from the plate
- Fix the biofilms with EtOH by adding 200 µL per well
- Incubate 15 min, RT, no shaking
- Remove the ethanol carefully and let the wells dry COMPLETELY (remove lid, takes about 30 min)
- Add 190 µL crystal violet (100X diluted) stain carefully without touching the walls of the wells
- Stain for 5 min, RT, no shaking
- Remove the stain carefully
- Wash the wells 2 times with MQ-water
- Let air dry (for 5-10 min)
- Solubilize the stain in 100% ethanol and incubate in RT for at least 1h (but no more than 3h) (I do 1h30)
- Measure absorbance at 595 nm

# Protocols UAntwerp

*Resazurin* assay *Staphylococcus* Biofilms in 96-well plates

**Keywords**

*S. aureus,S. epidermidis,* biofilm, resazurin

**Materials**

- 96-well plates, polystyrene, flat bottom (greiner bio-one BVBA, 655182) with *Staphylococcus* biofilm
- Automatic multichannel pipette
- Vacusafe 8-channel Vacuum pump system adapted for 96-well plates (afzuigtoestel bacterio)
- E.p. tips 2-200 µL, order number 0030.000.870 (= tips Vacusafe)
- E.p. tips 50-1250 µL, order number 0030.000.935 (= tips automatic multichannel pipette)
- Tecan
- Multistep pipette (eppendorf)

| Reference number | Firm | product | LMPH  number |
| --- | --- | --- | --- |
| 14190-094 | Gibco, lifetechnologies | PBS (sterile) |  |
|  |  | Resazurin 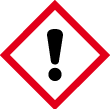 |  |
| Lab M, Lab 004 | International medical products | Trypton Soy Broth (TSB) |  |
|  |  |  |  |
|  |  |  |  |

**Methods**

- Discard the medium using the Vacusafe system (be careful you don’t touch the bottom of the wells in order to avoid disruption of the biofilm)
- Add 100 µL PBS to each well using an automatic 8-channel pipette
- Discard the PBS using the Vacusafe system (be careful you don’t touch the bottom of the wells in order to avoid disruption of the biofilm)
- Add 100 µL PBS to each well using an automatic 8-channel pipette
- Discard the PBS using the Vacusafe system (be careful you don’t touch the bottom of the wells in order to avoid disruption of the biofilm)
- Add 200 µL TSB to each well using an automatic 8-channel pipette
- Add 10 µL Resazurin to each well using a multistep pipette
- Incubate for 30 minutes at 37°C in the dark
- Measure fluorescence (λ_ex_ 550nm – λ_em_ 590nm)

**Remarks**

For 24-well plates use 1mL PBS/well, 2mL TSB/well and 100 µL Resazurin/well

Crystal Violet assay *Staphylococcus* Biofilms in 96-well plates

**Keywords**

*Staphylococcus*, biofilm, crystal violet

**Materials**

- 96-well plates, polystyrene, flat bottom (greiner bio-one BVBA, 655182) with *Staphylococcus* biofilm
- Automatic multichannel pipette
- Vacusafe 8-channel Vacuum pump system adapted for 96-well plates (afzuigtoestel bacterio)
- E.p. tips 2-200 µL, order number 0030.000.870 (= tips Vacusafe)
- E.p. tips 50-1250 µL, order number 0030.000.935 (= tips automatic multichannel pipette)
- Telix (Multiskan MCC 340 Microplate reader, Labsystems)
- Chemical Fume hood


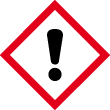


| Reference number | Firm | product | LMPH  number |
| --- | --- | --- | --- |
|  |  | PBS (non-sterile) |  |
| 20903.368 | VWR international | Technical Methanol 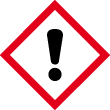 | 202 |
| 94448-  2.5L-F | Merck | Gram's Crystal Violet solution | 185 |
| MERC1.000 63.2500 | Merck, VWR | Glacial Acetic Acid | 443 |
|  |  | DemiQ (non-sterile) |  |

**Methods**

- Discard the medium using the Vacusafe system (be careful you don’t touch the bottom of the wells in order to avoid disruption of the biofilm)
- Add 100 µL PBS to each well using an automatic 8-channel pipette
- Discard the PBS using the Vacusafe system (be careful you don’t touch the bottom of the wells in order to avoid disruption of the biofilm)
- Add 100 µL PBS to each well using an automatic 8-channel pipette
- Discard the PBS using the Vacusafe system (be careful you don’t touch the bottom of the wells in order to avoid disruption of the biofilm)
- Add 150 µL methanol to each well using an automatic 8-channel pipette (in chemical fume hood polyvalent lab)
- Incubate for 15 minutes at room temperature
- Remove the methanol by turning upside down the plate above a suitable box in order to collect the methanol
- Remove the residual methanol by gently dabbing the plate on a green tissue
- Air dry plates in chemical fume hood
- In the meantime, you can prepare the 33% (v/v) Glacial Acetic Acid and 0,005% crystal violet solution
- Add 200 µL 0,005% crystal violet to each well using an automatic 8-channel pipette in chemical fume hood
- Incubate for 5 minutes at room temperature
- Remove the crystal violet by turning upside down the plate above a suitable box in order to collect the crystal violet
- rinse the plate under running tap water and collect the crystal violet through a funnel in an appropriate waste recipient
- Remove the residual crystal violet by gently dabbing the plate on a green tissue
- Air dry plates (the wells have to be dry completely)
- Add 250 µL 33% Glacial Acetic Acid to each well using an automatic 8-channel pipette
- Incubate for 15 minutes at room temperature
- Measure OD_570nm_ with telix (mix 45 seconds before measuring)

*Preparation of 0,005% (v/v) Crystal violet (fume hood):*

Dilute Gram's Crystal Violet solution 1:200 in non-sterile DemiQ Protect it from light by wrapping the bottle in tinfoil

*Preparation of 33% (v/v) Glacial Acetic Acid (fume hood):*

Add 330 mL Glacial Acetic Acid to 670 mL non-sterile DemiQ

**Remarks**

You can perform this assay in a non-sterile way

**S2. Control experiments data with Lab 5**

Data analysis was not performed for the treatment experiments as there was missing data necessary for the analysis. Including lab 5 data in the statistical analysis increases the reproducibility SD for all three methods. The biggest effect is observed for the plate count method, where the lab 5 dataset stands out as an outlier (~ 2.25 log difference from the mean). It was not possible to verify if the data was properly input in the datasheet due to lack of lab book records.

Summary of the control experiments results when data collected by Lab 5 were included.

**Table S2. Summary of analysis of the control data for the interlab protocol (ILP) with Lab 5 data included.**

| **Method** | **Mean Log ± SE** | **Units** | **Variance components** | | **Standard deviation** | |
| --- | --- | --- | --- | --- | --- | --- |
|  |  |  | Day + Error | Lab | Repeatability | Reproducibility |
| Plate count | 7.77 ± 0.56 | CFU/well | 3.3% | 96.7% | 0.25 | 1.37 |
| Resazurin | 0.56 ± 0.24 | µg/mL | 10.5% | 89.5% | 0.19 | 0.61 |
| Crystal Violet | 1.24 ± 0.19 | µg/mL | 7.7% | 92.3% | 0.13 | 0.48 |

**
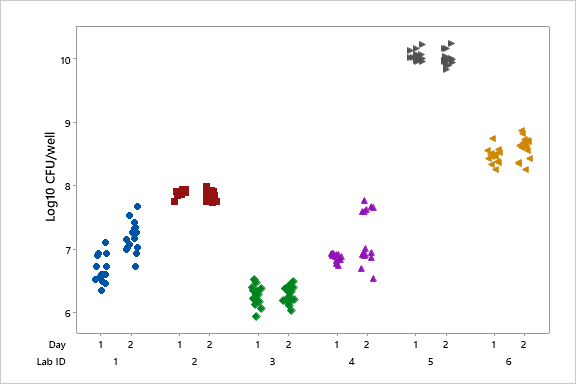
**

**Figure S2.1. Individual value plot of control experiment results for the plate count method with Lab 5 data included** Along the horizontal axis are listed the lab IDs and the two experimental days within each lab. Horizontal jitter has been applied to better visualize data points.

**
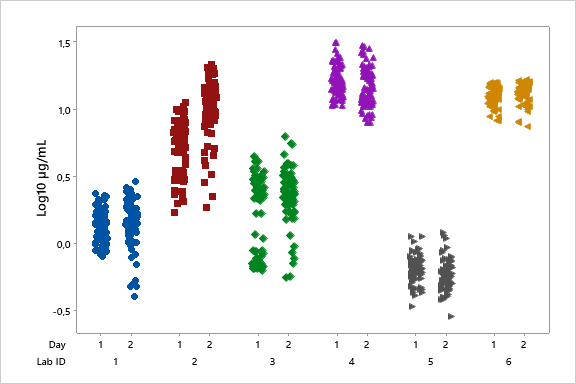
**

**Figure S2.2. Individual value plot of control experiment results for the resazurin method with Lab 5 data included** Along the horizontal axis are listed the lab IDs and the two experimental days within each lab. Horizontal jitter has been applied to better visualize data points.


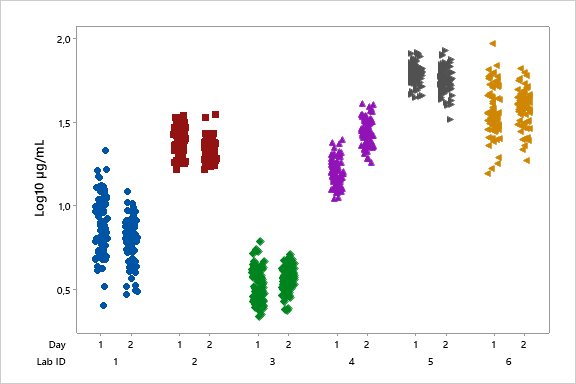


**Figure S2.2. Individual value plot of control experiment results for the crystal violet method with Lab 5 data included** Along the horizontal axis are listed the lab IDs and the two experimental days within each lab. Horizontal jitter has been applied to better visualize data points.

**S3. Plate reader test results**

Summary of the results for plate reader tests performed for crystal violet and resazurin. Standard curves and equations are shown for each individual laboratory.

**S3.1 Resazurin**

The fluorescence data collected from the plate reader test with the chemically reduced resazurin was used to plot the standard curves for each laboratory. Based on this data, a regression analysis was performed for the following:

- Fluorescence vs Resorufin concentration
- Log Fluorescence vs Log Resorufin concentration
- Log Fluorescence vs Resorufin concentration
- Fluorescence vs Log Resorufin concentration

Based on the regression analysis, Log Fluorescence vs Log Resorufin was chosen as the standard curve for the resazurin data. The residual plots (not shown) from the regression analysis indicated better fit of the Log vs Log model to the data, and better adherence to the modelling assumptions (normality and constant variance). This allows for better prediction of data points outside the range of the standard curve, which was necessary in the next steps of the statistical analysis.

**
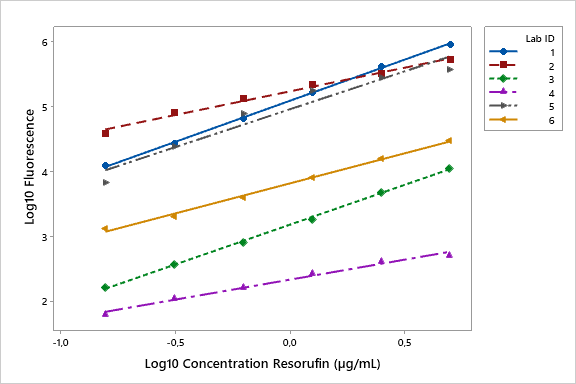
Figure S3.1. Scatter plot of Log Fluorescence vs Log Resorufin concentration for all 6 participating laboratories.**

The equations for each lab were generated by linear regression analysis and they were used to transform the fluorescence outputs into log resorufin concentration.

Equations per Lab

| **Lab ID** |  |  |  |
| --- | --- | --- | --- |
| **1** | Log Fluorescence | = | 5.0866 + 1.2650 Log Concentration |
| **2** | Log Fluorescence | = | 5.2335 + 0.7284 Log Concentration |
| **3** | Log Fluorescence | = | 3.1730 + 1.2260 Log Concentration |
| **4** | Log Fluorescence | = | 2.3248 + 0.6157 Log Concentration |
| **5** | Log Fluorescence | = | 4.9561 + 1.1667 Log Concentration |
| **6** | Log Fluorescence | = | 3.8140 + 0.9248 Log Concentration |

**S3.2 Crystal Violet**

The optical density (OD) data collected from the plate reader test was used to plot the standard curves as a function of crystal violet concentration for each lab. Regression analysis for the Absorbance vs Crystal violet concentration was performed.

**Figure S3.2. Scatter plot of OD vs Crystal violet concentration for all 6 participating laboratories.**

The equations for each lab were generated by linear regression analysis and were then used to transform the absorbance data to crystal violet concentrations. The crystal violet concentrations were further transformed from g/L to µg/mL.

Equations per Lab

| **Lab ID** |  |  |  |
| --- | --- | --- | --- |
| **1** | OD | = | 0.00485 + 106.70 Concentration |
| **2** | OD | = | -0.01042 + 145.04 Concentration |
| **3** | OD | = | 0.01581 + 108.60 Concentration |
| **4** | OD | = | 0.02924 + 82.41 Concentration |
| **5** | OD | = | 0.09051 + 119.00 Concentration |
| **6** | OD | = | 0.00662 + 84.23 Concentration |

**S4. Supplementary figures.**


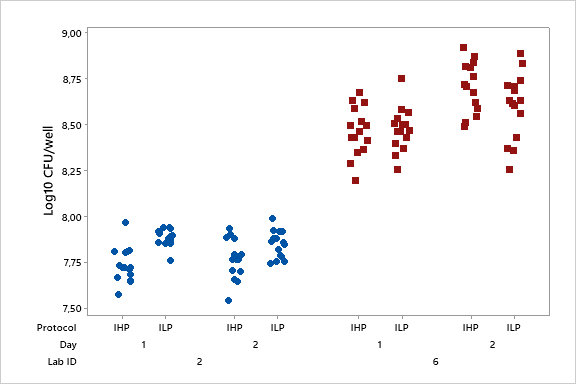


**Figure S4.1.** **Control experiment data comparing ILP and IHP protocols.** Each point in the graph is the log density (LD=log10(CFU/well)) of biofilm bacteria grown on a single well. Along the horizontal axis are listed the lab IDs, the two experimental days within each lab and the two protocols within each experimental day.


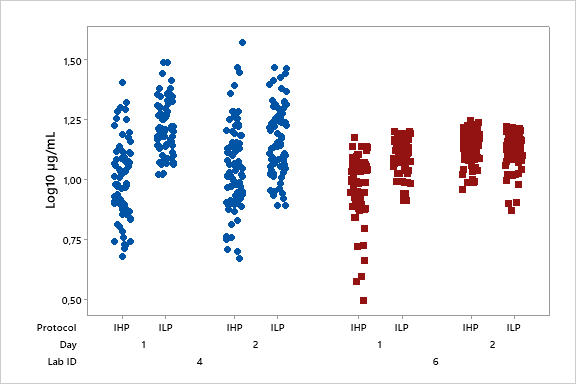


**Figure S4.2.** **Control experiment data comparing IHP and ILP protocols.** Each point in the graph is the log_10_ resorufin concentration (µg/mL) of biofilm bacteria grown on a single well. Along the horizontal axis are listed the lab IDs, the two experimental days within each lab and the two protocols within each experimental day.


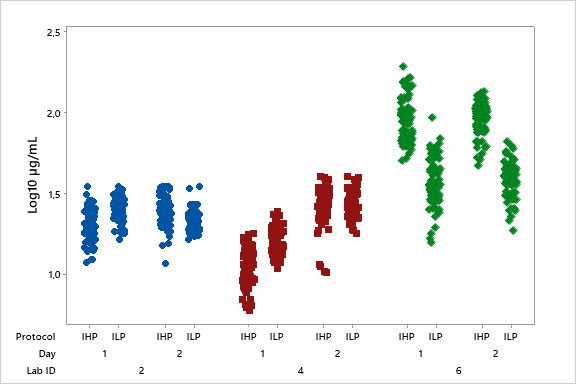


**Figure S4.3.** **Control experiment data comparing IHP and ILP protocols.** Each point in the graph is the log_10_ crystal violet concentration (µg/mL) of biofilm bacteria grown on a single well. Along the horizontal axis are listed the lab IDs, the two experimental days within each lab and the two protocols within each experimental day.

**
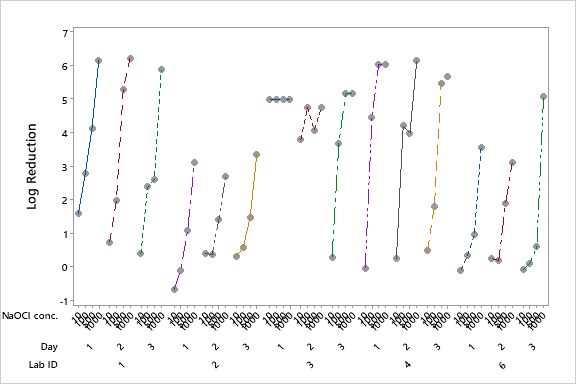
**

**Figure S4.4. Treatment experiment data for the plate count method.** Log reductions (LR) for the NaOCl treatment in the multi-lab study. The horizontal axis lists all lab IDs, the three experimental days and the 4 NaOCl concentrations tested. Each point in the figure is the mean LR for a single concentration of disinfectant in a single experiment.


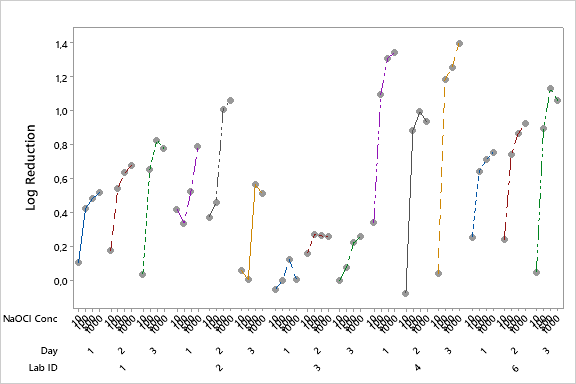


**Figure S4.5.** **Treatment experiment data for the resazurin method.** Log reductions (LR) for the NaOCl treatment in the multi-lab study. The horizontal axis lists all lab IDs, the three experimental days and the 4 NaOCl concentrations tested. Each point in the figure is the mean LR for a single concentration of disinfectant in a single experiment.


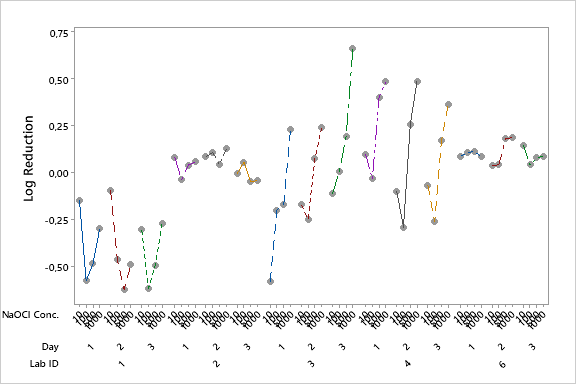


**Figure S4.6.** **Treatment experiment data for the crystal violet method.** Log reductions (LR) for the NaOCl treatment in the multi-lab study. The horizontal axis lists all lab IDs, the three experimental days and the 4 NaOCl concentrations tested. Each point in the figure is the mean LR for a single concentration of disinfectant in a single experiment.


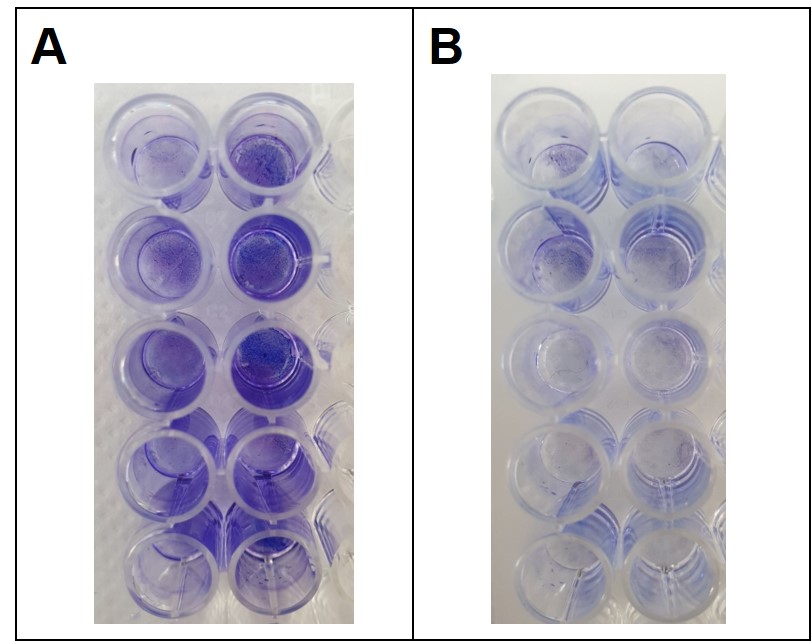


**Figure S4.7. Interaction between NaOCl and crystal violet.** Crystal violet staining of 96-well plates post incubation with different concentrations of NaOCl measured as Cl **A.** 2% vol/vol; **B.** 0.1 % vol/vol.

**D**

**C**

**B**

**A**


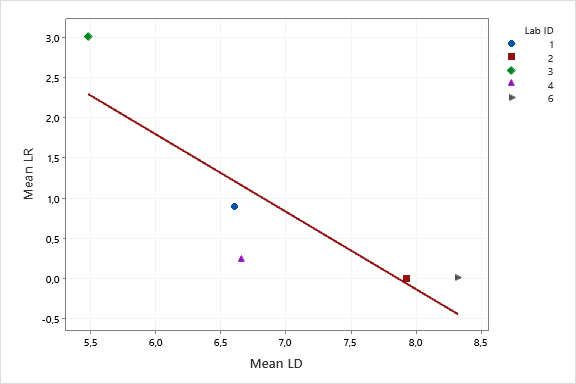

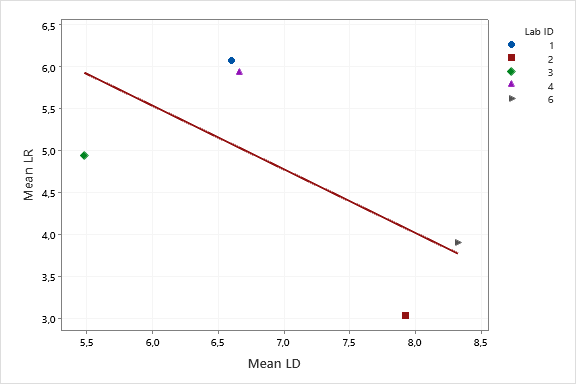

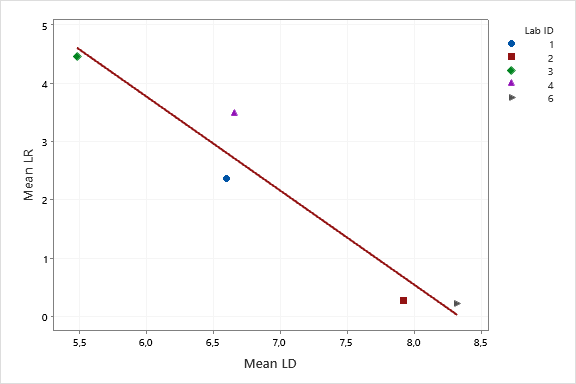

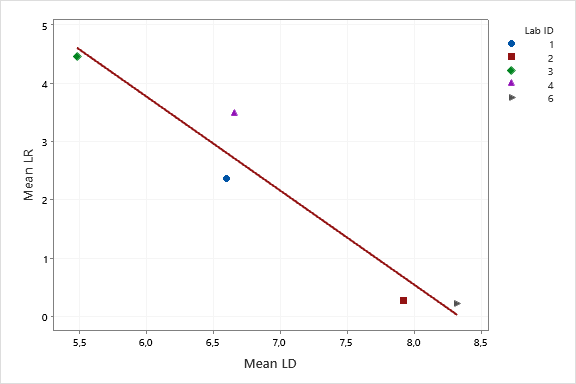


**Figure S4.8.** **Relationship between LR and LD in the treatment experiments for the plate count method.** Each data point represents the Mean LR and corresponding Mean LD for each lab at different concentrations of NaOCl: **A.** 10 mg/L **B.** 100 mg/L **C.** 500 mg/L **D.** 1000 mg/L
